# Midostaurin response in AML is shaped by a progenitor-like cell state selectively targeted by SMAC mimetics

**DOI:** 10.1038/s41698-026-01363-8

**Published:** 2026-03-11

**Authors:** Nona Struyf, Henrik Gezelius, Anders Lundmark, Chiara Barizza, Hidde Ploeger, Lucia Rico Pizarro, Mattias Vesterlund, Georgios Mermelekas, Albin Österroos, Anna Bohlin, Sofia Bengtzén, Kerstin Hamberg Levedahl, Rozbeh Jafari, Lukas M. Orre, Janne Lehtiö, Päivi Östling, Brinton Seashore-Ludlow, Jessica Nordlund, Sören Lehmann, Olli Kallioniemi, Tom Erkers

**Affiliations:** 1 Department of Oncology-Pathology, Karolinska Institutet, Science for Life Laboratory, Stockholm, Sweden; 2 Department of Medical Sciences, Science for Life Laboratory, Uppsala University, Uppsala, Sweden; 3 Department of Medical Sciences, Uppsala University Hospital, Uppsala, Sweden; 4 Department of Medicine, Center for Hematology and Regenerative Medicine, Karolinska Institute, Stockholm, Sweden; 5 Department of Public Health and Caring Sciences, Uppsala University, Uppsala, Sweden; 6 Institute for Molecular Medicine Finland, University of Helsinki, Helsinki, Finland

**Keywords:** Cancer, Drug discovery, Oncology

## Abstract

FLT3-mutated acute myeloid leukemia (AML) remains difficult to treat due to frequent resistance to FLT3 inhibitors like midostaurin. In this study, we observed a progenitor-like CD38^+^CD45RA^+^ leukemic cell population that may be associated with midostaurin resistance. Midostaurin-resistant cells display disrupted membrane architecture and a shift in signaling from STAT5 to PI3K/AKT, favoring survival over apoptosis. Functional drug testing was consistent with clinical response to midostaurin, and together with multi-omic profiling, including single-cell and proteomic analyses, indicated the presence and relevance of this resistant phenotype. Drug combination screening revealed that co-targeting with SMAC mimetics restores apoptotic competence and selectively depletes the resistant population when combined with midostaurin. In contrast, venetoclax combinations preferentially affected CD34^hi^ cells, underscoring distinct subpopulation vulnerabilities. These findings may point to a biologically relevant mechanism underlying midostaurin resistance.

## Introduction

Acute myeloid leukemia (AML) is a hematologic malignancy characterized by the clonal expansion of immature myeloid progenitors in the bone marrow, leading to impaired hematopoiesis. It is a heterogeneous disease with 5-year survival rates of 35–40% in patients under 60, and 15% for patients over 60, due to therapy resistance and high relapse rates^1^. One-third of all patients with AML carry an activating mutation in the FMS-like tyrosine kinase (FLT3) gene, which is associated with a poor prognosis and higher relapse rates. Recurrent aberrations in the FLT3 gene include internal tandem duplications (ITD) and point mutations in the tyrosine kinase domain (TKD). FLT3-ITD mutations, which typically occur in the juxtamembrane domain, are associated with constitutive receptor activation and confer a worse prognosis than FLT3-TKD point mutations, which occur in the activation loop and can be associated with resistance to certain inhibitors^2^. FLT3 mutations are among the most common in AML and frequently co-occur with mutations in NPM1, DNMT3A, RUNX1, and PML-RARA. According to current genetic risk classification, FLT3-ITD mutations are considered intermediate risk when not accompanied by adverse-risk mutations such as TP53 or RUNX1. In contrast to prior guidelines, the allelic ratio of FLT3-ITD no longer independently determines risk category, and risk assignment is now more strongly influenced by the presence or absence of co-occurring mutations^3^.

FLT3 mutations can be therapeutically targeted with FLT3 inhibitors (FLT3i), which are broadly classified into two generations. First-generation FLT3i, such as midostaurin and sorafenib, are multi-kinase inhibitors with activity against a range of tyrosine kinases, including KIT and VEGFR. Second-generation inhibitors, such as gilteritinib and quizartinib, are more selective for FLT3 and include both type I (active conformation-binding) and type II (inactive conformation-binding) compounds^4^. Midostaurin, a first-generation inhibitor, remains the only FLT3i currently approved for use in newly diagnosed FLT3-mutant AML (in combination with induction chemotherapy), while second-generation FLT3 inhibitors are primarily used in the relapsed/refractory setting^4,5^. In Sweden, all AML patients under 70 with FLT3-ITD or -TKD mutations receive standard induction therapy consisting of daunorubicin and cytarabine (DA 3 + 5) followed by midostaurin^6^. While midostaurin has improved outcomes for FLT3^mut^ patients, response rates of around 60% indicate that not all patients benefit from treatment, despite carrying the same mutation^5^. Quizartinib, a second-generation type II and highly selective FLT3i, was shown to be safe to use in combination with standard induction therapy in de novo FLT3^mut^ patients and showed improvement over standard induction therapy alone^7,8^. It has more recently been approved for de novo FLT3-ITD patients in combination with standard induction therapy. Although no head-to-head trials have been conducted yet, quizartinib demonstrated improved overall survival compared to chemotherapy alone in newly diagnosed FLT3-ITD patients in the QuANTUM-First trial (NCT02668653), suggesting potential as an alternative to midostaurin in this setting^7^. Another ongoing clinical trial is evaluating the addition of gilteritinib, a second-generation type I FLT3i, to standard induction therapy in comparison to midostaurin (HOVON 156, NCT04027309). Additionally, combination therapies have been under investigation, such as adding the Bcl-2 inhibitor venetoclax to current regimens or combining midostaurin, venetoclax, and the hypomethylating agent azacitidine in older, unfit FLT3^mut^ patients^9,10^. Although anti-apoptotic drugs have the potential to be used in combination with FLT3i, there is still limited understanding of the mechanisms underlying FLT3i resistance^9,11^. It is hypothesized that mutations in FLT3 and the dysregulation of downstream pathways have an impact on the anti-apoptotic proteins, and as a consequence, enable long-term survival of FLT3 mutant cells. This, in turn, also affects combination therapy approaches with anti-apoptotic drugs such as venetoclax, where the balance of anti-apoptotic proteins can affect drug response^9^.

Nevertheless, FLT3 inhibition is considered among the most selective strategies for targeting leukemic cells in FLT3^mut^ patients, emphasizing the need to identify strategies to overcome FLT3i resistance in this patient group^12^.

**Fig. 1:**
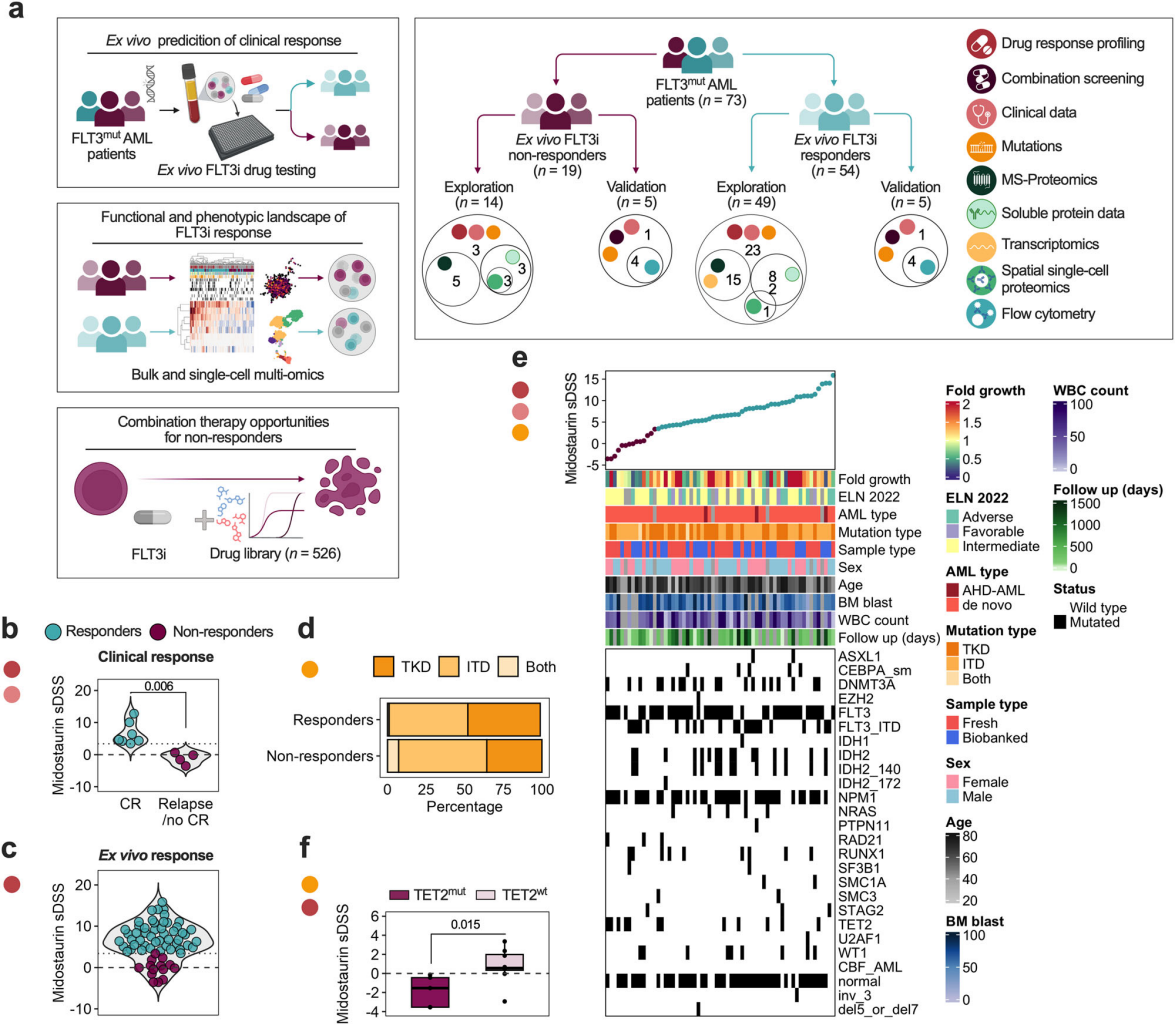
Ex vivo midostaurin response is in line with clinical outcome and TET2 mutation status. **a** Study overview. **b** Clinical outcome for patients treated with midostaurin (*n* = 11), statistical significance was assessed with the Mann–Whitney test, CR = complete remission after induction therapy. **c** Ex vivo midostaurin selective drug sensitivity score (sDSS) for all FLT3^mut^ patients (*n* = 63), a threshold of 3.46 (dashed line) was selected based on the clinical outcomes to divide patients into responders (*n* = 49) and non-responders (*n* = 14). **d** A visualization of mutation type showing distribution of FLT3-TKD- and -ITD in both groups. **e** An overview of clinical parameters, co-mutations and cytogenetic profiles of all FLT3^mut^ patients in relation to midostaurin sDSS. **f** A comparison of midostaurin sDSS between TET^mut^ and TET^wt^ non-responders, shown with the Mann–Whitney test.

In this study, we characterize the functional and molecular landscape associated with FLT3i response in FLT3^mut^ AML patients (Fig. 1a). We employed drug sensitivity and resistance testing (DSRT), a high-throughput ex vivo screening method where bone marrow (BM-MNCs) or peripheral blood (PBMCs) mononuclear cells are tested against 527 approved and investigational oncology drugs, including multiple FLT3i (Supplementary Fig. 1a, Supplementary Data 1)^13^. We profiled 63 FLT3^mut^ patients and complemented this with mass spectrometry (MS)-based proteomics, RNA-sequencing (seq), soluble proteome analysis, and spatial single-cell proteomics. The findings were corroborated by combination drug testing, flow cytometric drug sensitivity assays of cell subpopulations, and phospho-flow cytometry.

## Results

### Ex vivo midostaurin sensitivity aligns with observed clinical outcomes in patients receiving induction chemotherapy and midostaurin

We performed ex vivo DSRT on BM-MNCs or PBMCs from 63 FLT3^mut^ AML patients, including drugs that target FLT3. Each drug was tested in 5-point concentrations across a 10,000-fold concentration range. To evaluate whether ex vivo sensitivity to midostaurin reflects clinical outcomes, we examined the subset of patients (*n* = 11) who received standard induction chemotherapy (DA 3 + 5) followed by 14 days of midostaurin. Among these patients, those who achieved complete remission (CR) had significantly higher ex vivo midostaurin sensitivity compared to patients who failed to achieve CR or relapsed (*p* = 0.006; Fig. 1b). The lowest selective drug sensitivity score (sDSS) in patients achieving CR was 3.46 and was selected as a cutoff to define ex vivo responders versus ex vivo non-responders in the full cohort (Fig. 1c, hereon referred to as responders and non-responders, respectively). On average, responders exhibited a midostaurin half-maximal inhibitory concentration (IC_50_) of 145 nM, compared to 548 nM in non-responders. Drug response curves for non-responders closely resembled those of healthy BM donors, suggesting limited sensitivity (Supplementary Fig. 1b, Supplementary Data 2).

The prognostic relevance of FLT3 allelic ratio and mutation type (ITD vs. TKD) has been established in clinical trials^3,14^. However, in our cohort, 5-year overall survival was comparable between FLT3-ITD and FLT3-TKD patients (*p* = 0.7; Supplementary Fig. 1c). Furthermore, neither the mutation type nor allelic frequency was significantly associated with midostaurin sensitivity (Fig. 1d, Supplementary Fig. 1d–g). While high allelic burden predicted worse overall survival (*p* = 0.0067), it did not correlate with midostaurin response, suggesting that functional drug sensitivity may be a more direct predictor of treatment response than mutation status alone. Since FLT3-ITD and TKD samples exhibited similar ex vivo drug response patterns and both are managed with midostaurin in clinical practice, we combined them in our analyses to better understand molecular resistance and sensitivity mechanisms in FLT3^mut^ AML

To identify clinical or genetic features associated with midostaurin resistance, we performed multivariate analysis of clinical parameters and cytogenetic profiles. TET2 mutations were more common in non-responders (*p* = 0.063; Fig. 1e, Supplementary Tables 1–3), and patients harboring FLT3/TET2 co-mutations showed significantly lower midostaurin sDSS (*p* = 0.015; Fig. 1f), indicating that this subgroup may be intrinsically resistant to FLT3 inhibition.

### Ex vivo drug sensitivity profiling reveals broad functional divergence in FLT3-Mutated AML beyond FLT3 inhibitor response

**Fig. 2:**
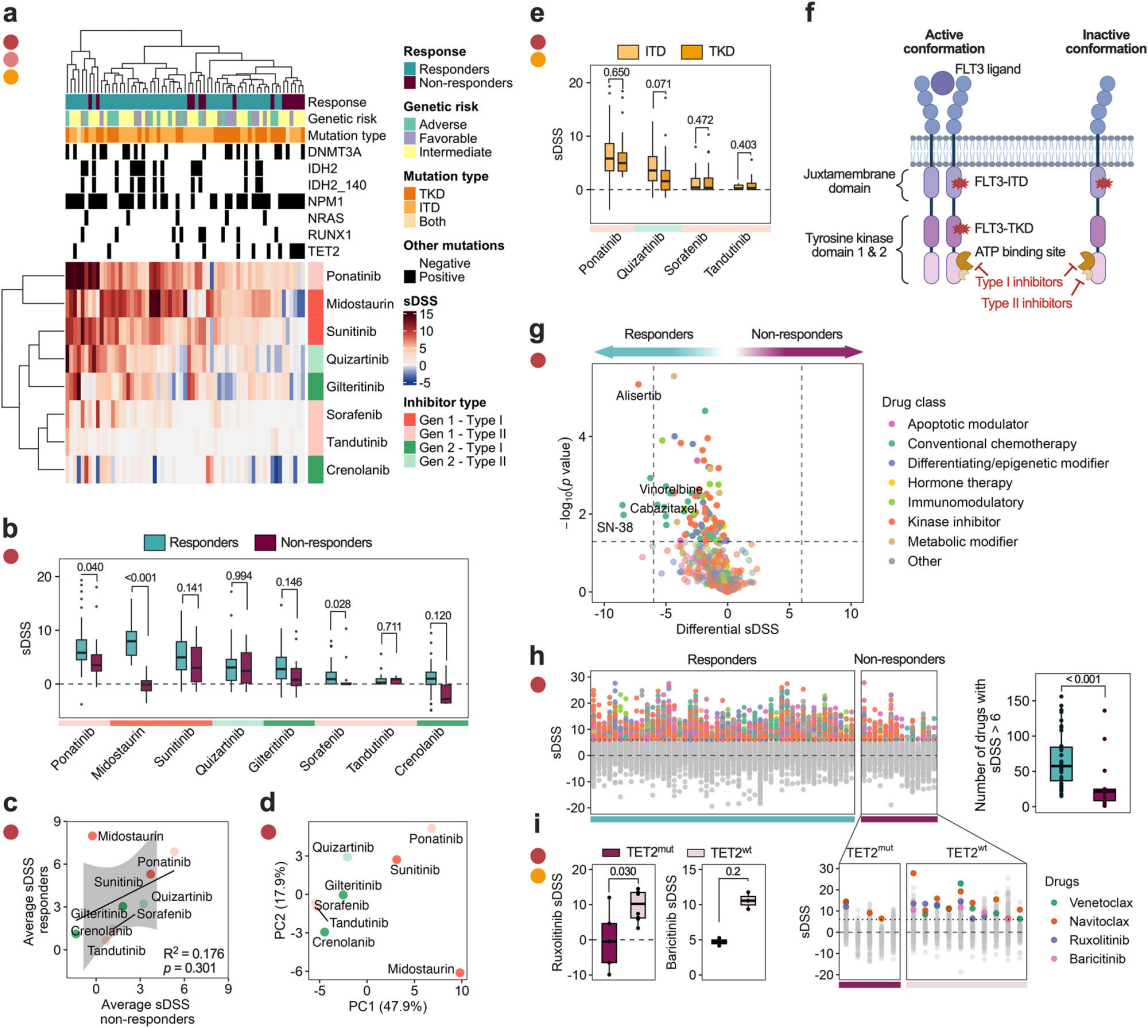
The drug response landscape in FLT3^mut^ AML shows polytherapy resistance in midostaurin non-responders, which is amplified in TET2 co-mutants. **a** A heatmap of FLT3i response in all FLT3^mut^ (*n* = 63) patients and the most common co-mutations, with hierarchical clustering performed on sDSS values. **b** Boxplots showing all sDSS values for all FLT3i in midostaurin responders (*n* = 14) and non-responders (*n* = 49) patients in the ordered by heatmap clustering. **c** *XY* plot showing average sDSS for all FLT3i in both groups, colored by inhibitor generation and type, with a linear regression model. **d** PCA plot showing the two largest sources of variance in FLT3i response in FLT3^mut^ (*n* = 63) patients. **e** A comparison of type II FLT3i response in FLT3-TKD- (*n* = 30) and -ITD (*n* = 33) patients. **f** Graphical representation of the FLT3 receptor and inhibitor binding sites. **g** Differential sDSS for all tested drugs (*n* = 527) between responders (*n* = 49) and non-responders (*n* = 14), where the 85th percentile of all drug responses was chosen as a cutoff (sDSS = 6), showing significantly different drugs (*p* < 0.05) above the cutoff, statistical significance was determined with a two-sample *t*-test with multiple testing correction using a BH-FDR of 5%. **h** Manhattan plot of sDSS values for all tested drugs in each patient, in responders and non-responders (left), and a comparison of the number of sensitive drugs in each group (right). **i** Boxplots showing difference in sDSS of selected drugs between TET2^mut^ (*n* = 5) and TET2^wt^ (*n* = 9) non-responders (left), and Manhattan plot for all tested drug responses in non-responders divided by TET2 mutation status, highlighting selected drugs (right). Statistical significance was assessed with the Mann–Whitney *U*-test unless otherwise specified.

To understand the variations in FLT3 inhibitor (FLT3i) response among FLT3^mut^ patients, we analyzed ex vivo DSRT data from 63 FLT3^mut^ AML patients. We observed that patients with high ex vivo midostaurin sensitivity also responded to other first-generation multi-kinase inhibitors, including ponatinib (type II) and sunitinib (type I) (Fig. 2a–c). Among the FLT3i tested, midostaurin showed ex vivo activity across a broader subset of patient samples, whereas second-generation FLT3i such as quizartinib and gilteritinib exhibited activity in more restricted subsets. This descriptive difference prompted further analysis of whether midostaurin sensitivity reflects on-target FLT3 pathway dependence or broader multi-kinase effects. To evaluate this, we derived a selective FLT3i sensitivity score based on the more selective second-generation FLT3i crenolanib, quizartinib, and gilteritinib. The score was defined as the mean sDSS across these compounds for each sample and scaled between 0 and 1 to facilitate cross-sample comparison. This composite score serves as an operational proxy for relative sensitivity to selective FLT3 inhibition, independent of midostaurin, rather than a direct measure of FLT3 pathway activation. We then assessed the relationship between this selective FLT3i score and ex vivo sensitivity to midostaurin, as well as to other broadly acting tyrosine kinase inhibitors with FLT3 activity, including sunitinib, ponatinib, sorafenib, and tandutinib. Midostaurin sensitivity exhibited only a weak and non-significant correlation with the selective FLT3i score (*r* = 0.16, *p* = 0.22), in contrast to ponatinib (*r* = 0.51, *p* < 0.001) and sunitinib (*r* = 0.60, *p* < 0.001), which showed moderate concordance. Sorafenib (*r* = 0.21, *p* = 0.095) and tandutinib (*r* = 0.32, *p* = 0.035) demonstrated weaker associations alongside overall lower ex vivo activity (Supplementary Fig. 2a).

Together, these results indicate that midostaurin sensitivity in this cohort is not strongly aligned with relative sensitivity to selective FLT3 inhibition, suggesting that its ex vivo activity cannot be explained solely by FLT3 pathway dependence and is likely influenced by compound-specific polypharmacology. Overall, inhibitor generation (first vs. second) appeared to explain more of the variation in drug response than binding conformation (type I vs. type II) (Fig. 2d). This pattern is consistent with, but does not directly demonstrate, differences in target selectivity and broader kinase inhibition profiles between earlier- and later-generation compounds. Despite type II inhibitors being designed to target FLT3-ITD mutations preferentially, we observed similar ex vivo sensitivity in both type I and II FLT3i in FLT3-ITD and -TKD samples (Fig. 2e, f). Together with the limited concordance between midostaurin sensitivity and selective FLT3 inhibitor response, these findings suggest that variation in ex vivo FLT3 inhibitor activity in this cohort cannot be readily explained by FLT3 mutation status or inhibitor binding mode alone.

Beyond FLT3i, midostaurin responders displayed greater overall sensitivity to other compounds in the high-throughput drug library (Fig. 2g). Using a stricter sDSS threshold due to variation in responses between drug classes at the 85th percentile of all drugs in all patients, responders had a median of 58 sensitive drugs, compared to 22 in non-responders (Fig. 2h). This suggests broad functional differences in between responders and non-responders that reach beyond genetics. While several drugs, such as venetoclax and ruxolitinib, were effective across both groups on average (Supplementary Fig. 2b), responses within the non-responder group were heterogeneous. BH3 mimetics (venetoclax, navitoclax) and JAK inhibitors (ruxolitinib, baricitinib) were more effective in TET2^wt^ midostaurin non-responders, but not in TET2^mut^ non-responders, supporting a model in which TET2 co-mutation amplifies therapy resistance (Fig. 2i, Supplementary Fig. 2c). Due to the limited number of TET2-mutant samples and lack of statistical power, only ruxolitinib showed significance while the other drugs indicate a trend. Thus, these findings warrant further investigation in a larger sample cohort.

### Midostaurin non-responders are enriched for immature phenotypes with distinct molecular signatures

**Fig. 3:**
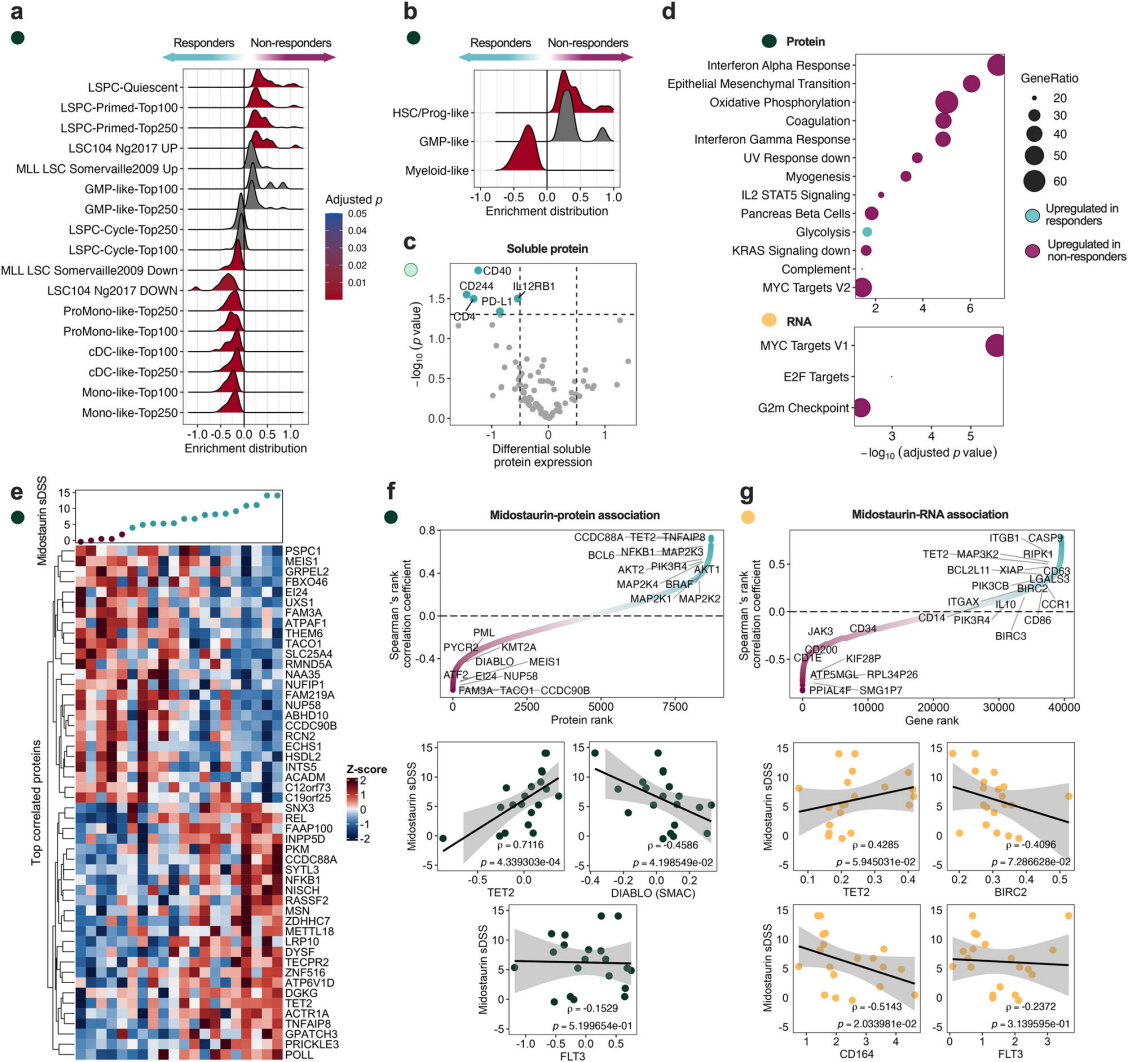
Midostaurin non-responders show enrichment for immature phenotypes with distinct molecular signatures. **a** and **b** Gene set enrichment analysis (GSEA) of MS-proteomics data using AML-specific gene sets showing enrichment distributions of each gene set and adjusted *p*-values. **c** Differential expression analysis of soluble proteins (*n* = 92) in responders (*n* = 11) and non-responders (*n* = 6), highlighting significant proteins (*p* < 0.05) with a differential normalized protein expression (NPX) above 0.5. Statistical significance was determined with a two-sample *t*-test with multiple testing correction using a BH-FDR of 5%. **d** GSEA of MS-proteomics and RNA-seq datasets (*n* = 20), using the MsigDB Hallmarks gene sets, comparing midostaurin responders (*n* = 15), and non-responders (*n* = 5), showing enrichment distributions of each gene set and adjusted *p* values. **e** A heatmap of the top 25 most positively and negatively significantly (*p* < 0.05) correlated proteins with midostaurin. **f** All tested proteins ranked by Spearman’s ranked correlation coefficient and *XY* plots of selected significant proteins, shown with a linear regression model. **g** All tested genes ranked by Spearman’s ranked correlation coefficient and *XY* plots of selected significant genes, shown with a linear regression model.

Next, to substantiate the underlying functional difference between responders and non-responders, we mapped the phenotypic and molecular landscape of FLT3^mut^ patients using MS-based proteomics, RNA-seq, and soluble protein data. Midostaurin non-responders displayed proteomic enrichment for gene sets that describe quiescent and primed leukemic stem and progenitor cells (LSPCs, Supplementary Data 3)^15,16^ (Fig. 3a). More specifically, non-responders were enriched for hematopoietic stem/progenitor cell phenotypes (HSC/Prog-like, LSPC-Quiescent, LSPC-Primed, LSC104 UP). In contrast, the responder patient cells showed enrichment for more differentiated phenotypes including conventional dendritic cell (cDC)- and monocyte-like^15^ cells (ProMono-, Mono-, cDC-, and Myeloid-like, Fig. 3b). Orthogonally, soluble protein data showed increased levels of CD40, CD244, PD-L1, CD4, and IL12RB1 in responders, demonstrating increased immune activity in this group (Fig. 3c). Gene set enrichment analysis (GSEA) using the MsigDB Hallmark^17^ gene set on both MS-proteomics and RNA-seq data revealed enrichment of Interferon-alpha and Interferon-gamma response sets and IL2-STAT5 signaling, consistent with a chronic, interferon-driven inflammatory state in non-responders, together with enrichment in canonical proliferation hallmarks E2F targets, MYC targets, and G2M checkpoint (Fig. 3d). Midostaurin non-responders exhibit a progenitor-like signature with increased inflammation and proliferation, prompting further investigation into the molecular drivers underlying this differential drug response.

Thus, we performed a linear regression to assess correlations between midostaurin sensitivity (sDSS) and protein/gene expression. Significant correlations were identified for 440 proteins and 1222 genes (Supplementary Data 4 and 5). Proteins such as TET2 (*ρ* = 0.712, *p* = 0.0004), a key regulator of myelopoiesis, as well as transcription-repressor BCL6 (*ρ* = 0.573, *p* = 0.0083) were positively associated with midostaurin response, whereas AML-related proteins PML (*ρ* = −0.391, *p* = 0.0884) and KMT2A (*ρ* = −0.455, *p* = 0.0439), as well as the anti-apoptotic protein (IAP) DIABLO/SMAC (*ρ* = −0.459, *p* = 0.0420), showed negative associations (Fig. 3e and f). Interestingly, FLT3 receptor (*ρ* = −0.153, *p* = 0.5200) protein levels showed no relation to midostaurin response.

In the transcriptomic dataset, TET2 (*ρ* = 0.428, *p* = 0.0594) was positively correlated, similar as in the protein data (Fig. 3g, Supplementary Fig. 3a). Interestingly, although TET2 mutations were associated with resistance, higher TET2 protein and transcript levels correlated with midostaurin sensitivity, suggesting that functional expression may partially compensate for mutational loss and influence treatment response. In addition, there was a positive correlation with markers indicating a shift in immune state involving both activation markers TLR6 (*ρ* = 0.541, *p* = 0.0137) and CD28 (*ρ* = 0.463, *p* = 0.0398), as well as immune regulator IL10RB (*ρ* = 0.478, *p* = 0.0330). BIRC2*/*cIAP1 (*ρ* = -0.410, *p* = 0.0729), another protein in the IAP family, showed a similar negative correlation as DIABLO. Notably, HSC markers and differentiation regulators such as CD164 (*ρ* = −0.514, *p* = 0.0203), PIR (pirin) (*ρ* = −0.803, *p* = <0.0001), MIR155HG (ρ = -0.692, *p* = <0.0001), CSF2 (*ρ* = -−0.547, *p* = 0.0125), and IL7 (*ρ* = −0.532, *p* = 0.0157) were negatively correlated with midostaurin sensitivity. In line with proteomics, gene expression of the FLT3 receptor (*ρ* = −0.237, *p* = 0.3140) showed no relation to midostaurin response. This lack of correlation between FLT3 protein/gene expression and midostaurin response supports the notion that sensitivity is not solely dependent on FLT3 receptor abundance, but may be influenced by downstream pathway states, lineage priming, and apoptotic thresholds.

GSEA further revealed that genes and proteins negatively correlated with midostaurin response were enriched in proliferation-related pathways, while STAT3, TNF, and PI3K signaling pathways were positively associated. (Supplementary Fig. 3b and c). Together, these results indicate that midostaurin sensitivity is shaped by distinct signaling and cell states, independent of FLT3 receptor abundance, prompting further investigation into phenotypic markers, membrane organization, and pathway activity.

Finally, we assessed which molecular pathways correlated with the selective FLT3i sensitivity score and compared them with those associated with midostaurin response. The selective FLT3i score was positively associated with several signaling pathways that also correlated with midostaurin sensitivity, including MYC targets, PI3K/MTOR signaling, and STAT5-related signatures (Supplementary Fig. 3d and e). This overlap suggests partial convergence in downstream signaling associations despite differences in compound selectivity.

Notably, the degree of overlap between selective FLT3i- and midostaurin-associated pathways was higher at the transcriptomic level compared to the proteomic level (Supplementary Fig. 3f). While related signaling themes were observed in both data types, the specific pathway annotations differed, potentially reflecting differences in how PI3K/MTOR-related processes are captured at the transcriptomic versus protein level rather than discrete pathway activation states. Consistent with observations for midostaurin, the selective FLT3i score did not correlate with FLT3 protein abundance (*ρ* = −0.1594, *p* = 0.5005) or FLT3 gene expression (*ρ* = 0.015, *p* = 0.9518; Supplementary Fig. 3g and h).

### Midostaurin non-responders have increased CD45RA expression in CD38^+^ cells

**Fig. 4:**
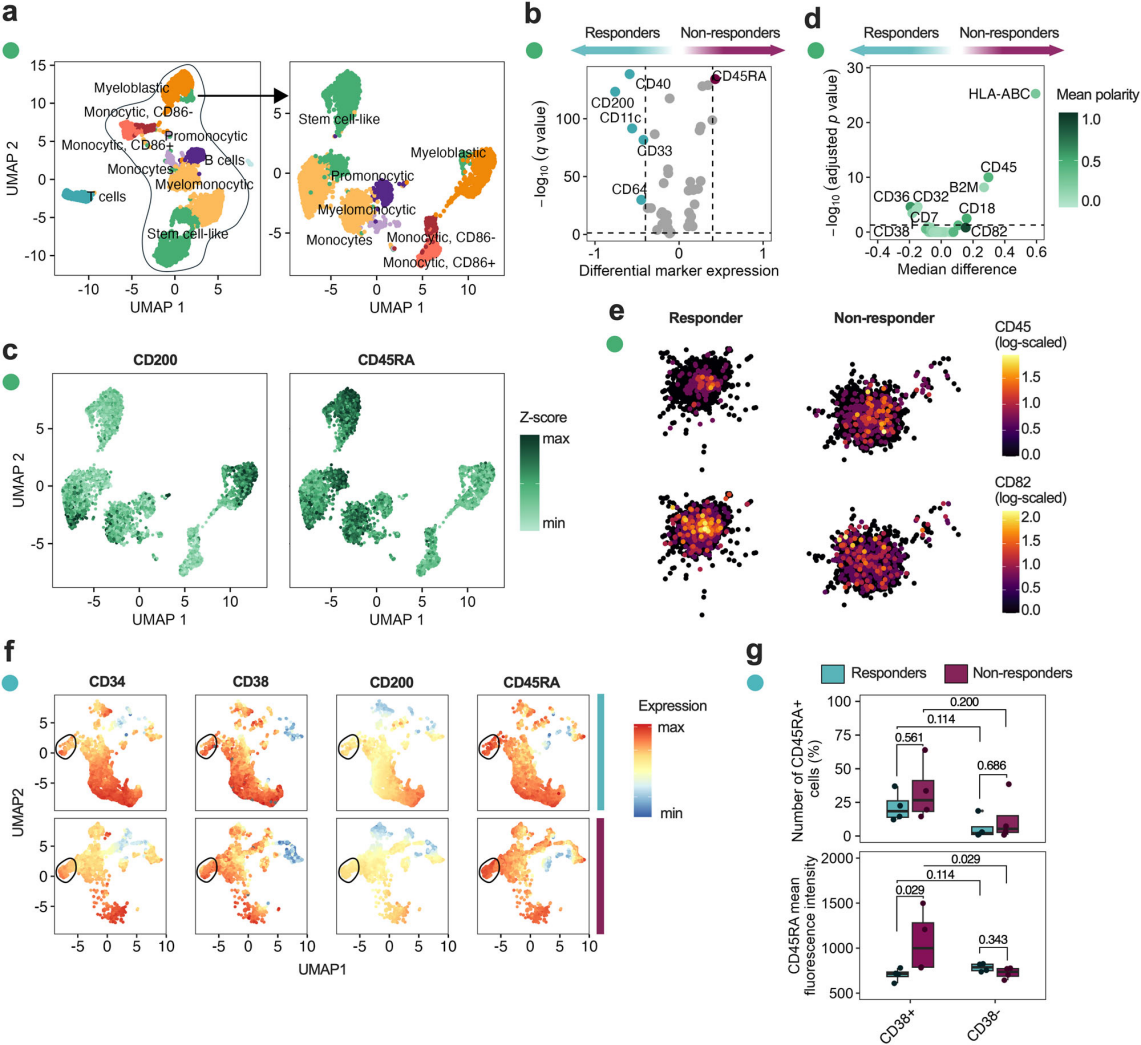
Midostaurin non-responders show increased CD45RA expression in CD38^+^ leukemic cells. **a** A UMAP showing single-cell clusters for single-cell spatial proteomics of midostaurin non-responders (*n* = 3) and responders (*n* = 3), clustered with the Louvain algorithm and annotations based on expression of surface markers (left), followed by a re-clustered UMAP of myeloid cells after gating out B and T cell clusters (right). **b** Differential expression analysis of surface markers measured by single-cell spatial proteomics showing significant surface proteins (*p* < 0.05) with a log_2_FC > 0.5. **c** UMAP of all myeloid cell clusters with overlaid expression of CD45RA and CD200, shown as normalized *Z*-scores. **d** Differential polarization of surface markers, showing markers colored by mean polarity with a significance cutoff (*p* < 0.05). **e** 2D visualization of spatial differences in CD45 and CD82 expression of two representative cells, shown as log-scaled counts for each marker. **f** UMAPs of flow cytometric validation in responder (*n* = 4) and non-responder (*n* = 4) cells, shown as relative marker expression where the CD45RA^+^CD38^+^CD34^low^ population has been highlighted in the black circle. **g** A comparison of the number of CD45RA^+^ cells and mean fluorescence intensity of CD45RA in CD38^+^ and CD38^−^ cells in responders (*n* = 4) and non-responders (*n* = 4). Statistical significance was assessed with the Mann–Whitney *U* test unless stated otherwise.

Based on the observed molecular difference between midostaurin responders and non-responders, we investigated whether altered expression of leukemic surface markers and membrane organization contributes to FLT3i response. We profiled 76 surface markers (Supplementary Data 6) using spatial single-cell proteomics^18^ in a subset of FLT3^mut^ patients, identifying 13 annotated immune and myeloid cell clusters (Fig. 4a, Supplementary Fig. 4a). Responders displayed increased expression of markers associated with committed myeloid progenitors (CD33, CD64, and CD11c), antigen presentation (CD40), and CD200. In contrast, non-responders were enriched for cells expressing the stem/progenitor marker CD45RA (Fig. 4b and c).

In addition to changes in surface marker expression, non-responders demonstrated altered membrane protein organization, including increased polarization of CD45 and CD82 (Fig. 4d). Co-localization analysis revealed reduced spatial association between CD82 and CD45, as well as with other signaling-associated markers, suggesting a reorganization of membrane microdomains in non-responders (Supplementary Fig. 4b). Notably, the increased polarization but reduced co-localization of CD45 and CD82 may reflect disrupted membrane architecture that could influence phosphatase accessibility or receptor clustering, potentially contributing to altered signaling dynamics observed in resistant cells (Fig. 4e).

To validate the phenotypic differences distinguishing midostaurin responders from non-responders, we examined an independent FLT3^mut^ patient cohort (4 responders, 4 non-responders) using flow cytometry. This panel included key markers identified in the spatial proteomics analysis, along with CD34 to improve resolution of classical leukemic stem cell (LSC) subsets (Supplementary Fig. 4c; Supplementary Table 4). Responders exhibited higher frequencies of CD34 (*p* = 0.029) and CD38 (*p* = 0.343), and mean fluorescence intensities (MFI) of CD64 (*p* = 0.2), CD38 (*p* = 0.029), and CD34 (*p* = 0.029), consistent with a trend towards a more differentiated but still precursor myeloid phenotype (Supplementary Fig. 4d and e). CD200 (*p* = 0.343) and CD45RA (*p* = 0.486) frequencies were also higher in this group; however, the MFI of CD45RA was notably greater in non-responders (Supplementary Fig. 4f), particularly within CD38⁺ (*p* = 0.029) cells (Fig. 4f and g).

These findings suggest that although CD45RA is expressed across both groups, its elevated intensity in CD38⁺ cells in non-responders supports the presence of a CD34-independent progenitor-like subset. This immature CD45RA⁺CD38⁺ population may contribute to functional drug resistance in a subset of FLT3^mut^ AML patients.

### Midostaurin resistance is associated with a signaling shift from STAT5 to AKT

**Fig. 5:**
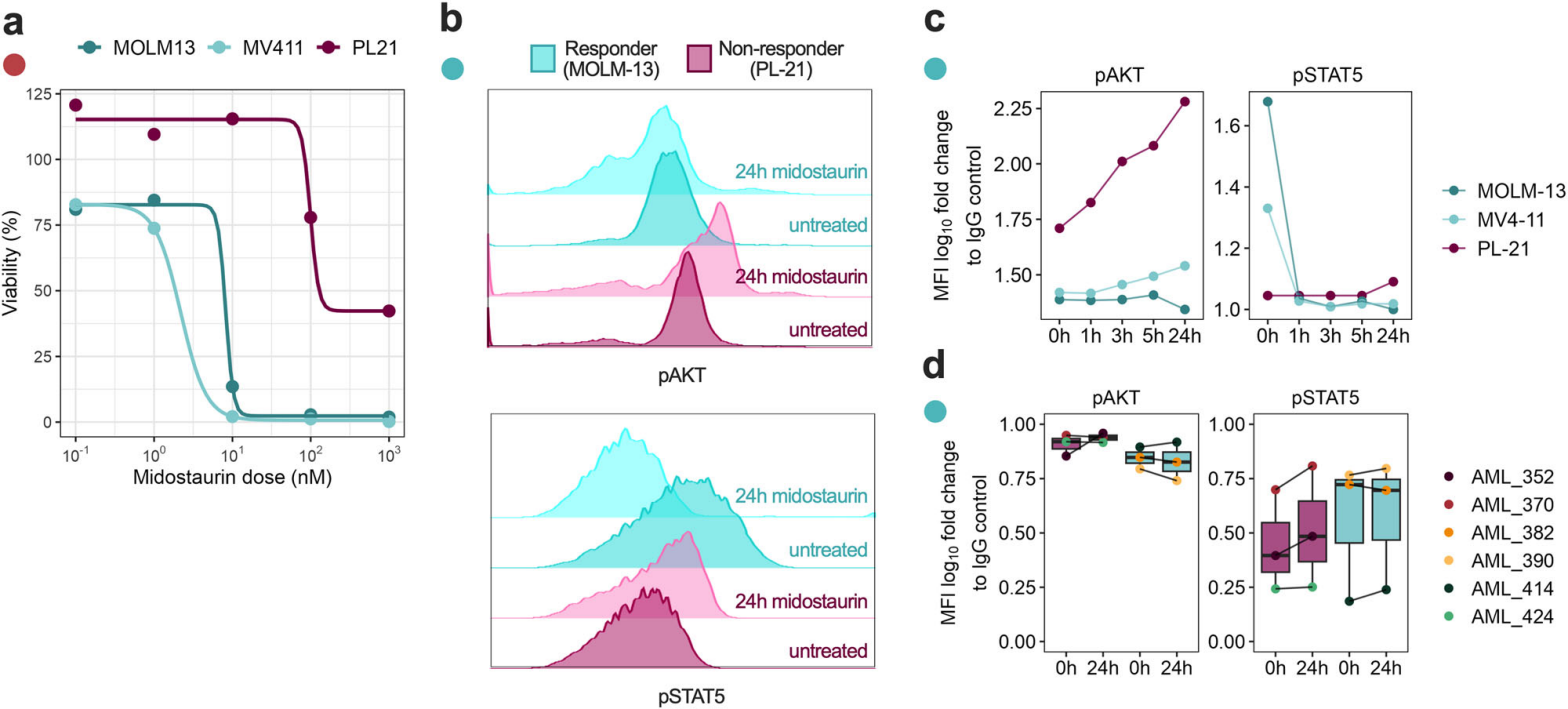
Non-responders show a signaling shift from STAT5 to AKT upon midostaurin treatment. **a** Drug–response curves showing the midostaurin sensitivity for all three FLT3^mut^ cell lines. **b** Histograms showing phosphorylation of pAKT and pSTAT5 measured by phosphoflow in the FLT3^mut^ cell lines PL-21 (non-responder) and MOLM-13 (responder) after 24 h treatment with midostaurin. **c** pAKT and pSTAT5 MFI measured at several timepoints from midostaurin stimulation in FLT3^mut^ cell lines PL-21 (non-responder), MOLM-13 (responder), and MV4-11 (responder), shown as log10 fold change to IgG control. **d** Boxplots showing log10 fold change to IgG control for pAKT and pSTAT5 MFI in responder (*n* = 3) and non-responder (*n* = 3) patient cells.

Given the observed enrichment of immature CD45RA⁺CD38⁺ cells and altered membrane organization in midostaurin non-responders, we next investigated whether these phenotypic differences were reflected in downstream signaling dynamics. Since CD45 and CD82 are known to modulate phosphatase activity and receptor clustering, we hypothesized that their spatial reorganization might influence key FLT3 signaling pathways. To test this, we performed phospho-flow cytometry to assess activation of MAPK/ERK, JAK/STAT5, and PI3K/AKT pathways in FLT3^mut^ AML cells following midostaurin treatment. Here, we used three FLT3^mut^ cell lines, of which MOLM-13 and MV4-11 were midostaurin responders, and PL-21 was a non-responder (Fig. 5a). Indeed, we observed distinct signaling dynamics between a responding and non-responding cell line upon midostaurin treatment. After 24 h, PL-21 exhibited sustained or increased phosphorylation of AKT, while MOLM-13 showed early and consistent dephosphorylation of STAT5 (Fig. 5b). Notably, STAT5 dephosphorylation was evident within one hour in MOLM-13 and MV4-11, whereas pAKT progressively increased in PL-21 over time (Fig. 5c). Similar phosphorylation pattern trends were observed in primary patient cell samples, although the sample size was too limited for statistical analysis (Fig. 5d).

These findings suggest that in non-responders, midostaurin possibly fails to adequately suppress the PI3K/AKT axis, potentially enabling survival signaling that may contribute to drug resistance.

### Midostaurin demonstrated synergy with SMAC mimetics in preclinical models of resistant cells

**Fig. 6:**
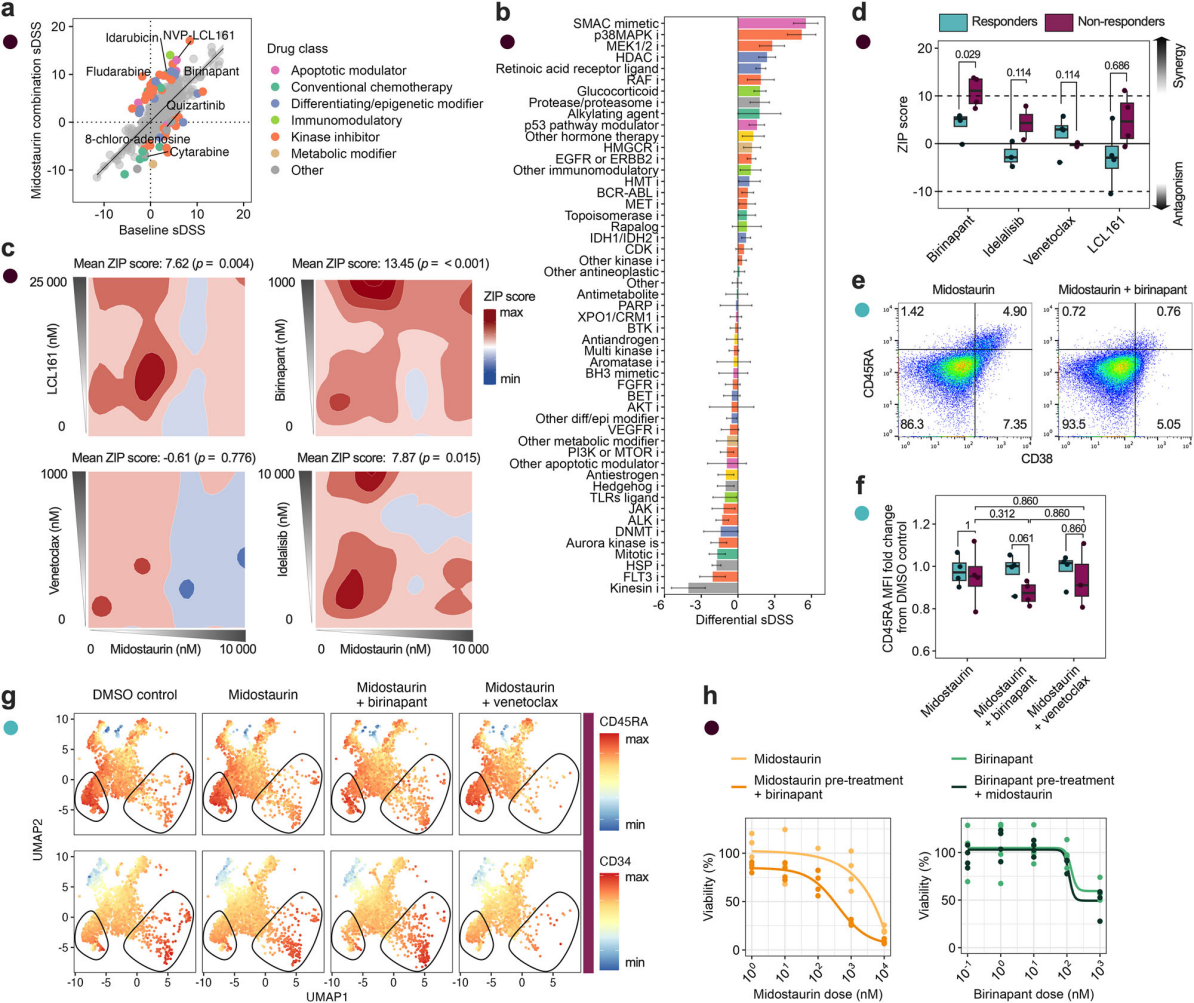
Midostaurin synergizes with SMAC mimetics in immature CD38^+^CD45RA^+^ cells. **a** Combination screening on non-responder patient cells (*n* = 1) displaying the selective drug sensitivity score (sDSS) for all tested drugs (*n* = 526), showing drugs with a differential sDSS > 4 for visualization purposes, colored by drug class, the baseline sDSS on the *x*-axis, and combination with 1200 nM midostaurin on the *y*-axis. **b** A waterfall plot with the differential sDSS for all drug subclasses tested during combination screening, shown as drug subclass mean with SEM and ranked by mean and colored by drug class. **c** Synergy screening of midostaurin with selected drugs, shown for one representative non-responder patient with mean ZIP scores and *p* values. **d** Synergy screening results for all responders (*n* = 4) and non-responders (*n* = 4) showi*n*g mean ZIP scores for all tested combinations where ZIP > 10 equals synergy, ZIP between 10 and −10 is additive, and ZIP < −10 equals antagonism. **e** Dot plot of a representative non-responder patient of CD38 and CD45RA expression measured by flow cytometry, showing midostaurin (1000 nM) alone and in combination with birinapant (280 nM). **f** Boxplots showing CD45RA MFI fold change from DMSO control after 72 h treatment with midostaurin (1000 nM) alone or in combination with birinapant (100 nM) and venetoclax (10 nM), measured by flow cytometry in responders (*n* = 4) and non-responders (*n* = 4). **g** UMAPs showing CD45RA and CD34 expression in non-responder (*n* = 4) cells for midostaurin (1000 nM) alone or in combination with birinapant (100 nM) and venetoclax (10 nM). **h** Sequential drug treatment of midostaurin (1200 nM, left) and birinapant (280 nM, right) for 24 h followed by a 5-dose response of the other drug for 48 h in PL-21, performed in quadruplicate. Statistical significance was assessed with the Mann–Whitney *U* test unless otherwise specified.

Given the persistent activation of the PI3K/AKT pathway in midostaurin non-responders, the enrichment of immature CD45RA⁺CD38⁺ phenotypes, and the altered expression of apoptosis-regulating proteins and genes observed in our molecular profiling, we hypothesized that these cells may exhibit a primed apoptotic state. To explore therapeutic strategies capable of exploiting this vulnerability, we performed combinatorial drug screening across a library of 526 compounds, including a subset of apoptotic modulators, using primary FLT3^mut^ AML cells from a non-responder, as well as the inherently FLT3i-resistant FLT3^mut^ PL-21 cell line (Fig. 6a, Supplementary Fig. 5a).

Among the tested drug classes, apoptotic modulators indeed showed the strongest enhancement in drug sensitivity in the patient sample, followed by differentiating/epigenetic modifiers (Supplementary Fig. 5b). Similar results were observed in PL-21 cells, with apoptotic modulators showing consistent effects. Across both models, SMAC mimetics emerged as the most effective subclass (Fig. 6b; Supplementary Fig. 5c). Other active subclasses included MEK1/2 inhibitors and retinoic acid receptor ligands. The patient cells also responded to HDAC, p38 MAPK, and PI3K inhibitors, while PL-21 cells showed sensitivity to PI3K/AKT inhibitors. In contrast, JAK inhibitors displayed reduced sensitivity in combination with midostaurin in the patient cells, while both kinesin and aurora kinase inhibitors showed reduced sensitivity across both models.

From these results, we selected four drugs for synergy testing in eight primary AML samples: the SMAC mimetics birinapant and LCL161, the PI3K inhibitor idelalisib, and the BH3 mimetic venetoclax. These drugs were chosen based on the combination drug screening, as well as clinical relevance. Among these, birinapant showed the highest synergy with midostaurin, with significantly elevated synergy in non-responders (*p* = 0.029; Fig. 6c and d, Supplementary Fig. 5d). LCL161 also showed partial synergy in some non-responder samples. Idelalisib and venetoclax exhibited additive but not synergistic effects with midostaurin in non-responders. Similarly, the combinations with LCL161 and birinapant restored the % inhibition of the non-responders to levels on par with the responders, while venetoclax and idelalisib did not (Supplementary Fig. 5e and f).

To understand whether this synergy was linked to leukemic cell phenotype, we performed flow cytometry to profile drug effects on distinct subpopulations. Interestingly, the combination of midostaurin and birinapant preferentially depleted CD38^+^CD45RA^+^ cells, the subset previously found to be enriched in non-responders (Fig. 6e). Notably, this combination also reduced CD45RA expression (MFI) more effectively than midostaurin monotherapy or other combinations (Fig. 6f and g, Supplementary Fig. 6a and b). While combinations with venetoclax and idelalisib also lowered CD45RA MFI, these predominantly targeted CD34^hi^ cells, suggesting the loss of CD45RA in these settings is likely secondary to depletion of CD34^+^CD45RA^+^ population (Supplementary Fig. 6c).

Then, to further investigate the biological basis of midostaurin resistance and its modulation, we examined whether drug scheduling influences apoptotic susceptibility in resistant AML cells. Sequential treatment with midostaurin followed by SMAC mimetics resulted in greater cell depletion than either monotherapy or reverse-order combinations (Fig. 6h; Supplementary Fig. 6d). These findings suggest that midostaurin may alter apoptotic signaling dynamics, potentially priming resistant cells for enhanced IAP inhibition.

To contextualize these observations within clinically relevant treatment frameworks, we modeled the effects of cytarabine, a standard first-line agent in AML. While cytarabine followed by midostaurin did not reduce cell viability in non-responder samples, the addition of SMAC mimetics, either alone or in combination with midostaurin, led to substantial reductions in viability (Supplementary Fig. 6e). These results support a mechanistic model in which sequential perturbation of survival and apoptotic pathways can overcome resistance in immature FLT3^mut^ AML cells.

### Midostaurin sensitivity is associated with differentiation state in both FLT3^mut^ and FLT3^wt^ AML

Given the clinical exploration of FLT3i in patients without FLT3 mutations, such as in the QuANTUM-WILD trial (NCT06578247), we assessed whether the phenotypic predictors of midostaurin response identified in FLT3^mut^ AML extend to patients without FLT3 mutations. We analyzed a cohort of FLT3^wt^ AML samples using integrated drug testing and proteomic profiling (Supplementary Fig. 7a, Supplementary Tables 5–7). After applying a similar cutoff for midostaurin response, we found that responses to midostaurin mirrored those of other FLT3i across both genotypes, though FLT3^mut^ patients generally exhibited greater overall sensitivity (Supplementary Fig. 7b–e). Analysis of cellular phenotypes based on protein expression revealed that FLT3^mut^ patients were enriched for immature, LSPC-like profiles, whereas FLT3^wt^ patients were enriched for mature myeloid and GMP-like states (Supplementary Fig. 7f and g). Notably, when stratifying by midostaurin response rather than genotype, we observed consistent trends. Non-responders, independent of mutation status, showed enrichment for LSPC-associated proteins, while responders exhibited enrichment of markers associated with mono-like proteins and more differentiated states (Supplementary Fig. 7h). These findings suggest that the differentiation state of AML cells may be a determinant of FLT3i sensitivity in addition to FLT3 mutation status alone, and that phenotypic profiling could complement genetic testing in predicting FLT3i response, supporting the rationale behind ongoing clinical trials evaluating FLT3 inhibitors in FLT3^wt^ AML.

## Discussion

FLT3-mutated AML remains a clinically heterogeneous disease, with therapeutic resistance continuing to limit the efficacy of FLT3-targeted agents. In this study, we employed a multi-omic approach integrated with ex vivo drug-sensitivity profiling to dissect the phenotypic and molecular landscape of FLT3 inhibitor response.

Consistent with recent updates to the ELN risk classification, which now considers FLT3-ITD an intermediate-risk mutation due to the demonstrated clinical benefit of midostaurin, our data show no clear functional distinction between FLT3-ITD and FLT3-TKD subtypes. This supports clinical trial findings where both subtypes appear to benefit comparably from midostaurin^5^. Similarly, we found no clear association between FLT3i response and co-occurring NPM1 or CBF mutations, despite their known favorable prognostic impact in chemotherapy-based regimens. Thus, while allelic burden and co-mutations may retain prognostic value for chemotherapy outcomes, they do not serve as biomarkers for FLT3i sensitivity, indicating the need for other companion diagnostics.

In line with other functional precision medicine studies^19–21^, our findings indicate that ex vivo drug testing may reflect clinical response. The number of patients with clinical outcome data in our study is, however, limited because of strict inclusion criteria, and only a small portion of patients were included after the introduction of midostaurin into the clinical routine. Three of the clinical non-responders initially responded when receiving intensive chemotherapy induction in combination with midostaurin, but the responses were brief and likely stemmed from induction chemotherapy rather than from midostaurin itself. This could have immediate implications for treatment stratification and encourages further studies to validate functional profiling in FLT3^mut^ AML management. Although the sDSS cutoff for midostaurin response may seem low, this likely reflects the reduced ex vivo sensitivity of tyrosine kinase inhibitors in the presence of HS-5 conditioned medium, an established model that more closely mimics the BM microenvironment^22^. Importantly, we observed reproducible differences in ex vivo drug responses between midostaurin responders and non-responders, even within a relatively small patient cohort, and across different experimental platforms.

Our data suggest the presence of a progenitor-like leukemic cell state that may contribute to midostaurin resistance. This population exhibits altered membrane organization and a signaling shift from STAT5 to PI3K/AKT, consistent with a phenotype that could favor survival signaling. Importantly, this resistant cell state was broadly detectable in bulk transcriptomic or proteomic analyses and emerged consistently across high-resolution single-cell platforms, including spatial proteomics and phospho-flow cytometry. These findings build on prior observations linking FLT3 mutations to impaired myeloid differentiation but go further by pinpointing a specific immature phenotype associated with FLT3i resistance. Emerging literature also recognizes that traditional leukemic stem cell markers such as CD34 and CD38 may not fully capture the heterogeneity and plasticity of leukemia-initiating cells and supports integrating CD45RA and CD200^16,23–25^.

TET2 co-mutations were more prevalent in non-responders and may further contribute to differentiation blockade and the persistence of leukemic progenitors with high self-renewal capacity, highlighting the complexity of genetic and phenotypic factors in shaping midostaurin response^26^. Together, our data provide a step forward in understanding FLT3i resistance and highlight the need for refined diagnostic and therapeutic strategies targeting leukemic cell-state heterogeneity.

Moreover, we observe that these cell state features are associated with a divergence in downstream signaling between midostaurin responder and non-responder cells. Midostaurin-sensitive samples exhibited rapid STAT5 dephosphorylation, consistent with the established mechanism of FLT3 inhibition^27^. In contrast, non-responders displayed sustained AKT phosphorylation and a shift toward PI3K/AKT-associated survival signaling. This pattern was further supported by MS-based proteomic analyses showing altered expression of apoptosis-regulating proteins, including SMAC/DIABLO and cIAP1/2. These inhibitors of apoptosis proteins (IAPs) function downstream of PI3K/AKT signaling and contribute to apoptotic evasion. Their upregulation in samples with low ex vivo midostaurin response is consistent with a model in which persistent AKT activity may stabilize IAPs, thereby suppressing caspase activation and promoting cell survival. SMAC mimetics antagonize IAPs, restoring apoptotic competence^28^. Accordingly, the observed synergy between midostaurin and the SMAC mimetic birinapant may reflect a coordinated disruption of survival and apoptotic pathways, potentially mitigating resistance associated with sustained PI3K/AKT signaling.

Furthermore, our spatial proteomics data provide a valuable bridge between our phenotypic observations and signaling behavior in midostaurin responders and non-responders. CD200 is associated with immune suppression and inhibition of MAPK and PI3K/AKT signaling, suggesting it contributes to a more immunosuppressive yet differentiation-prone phenotype^29,30^. Conversely, CD45RA may influence STAT5 signaling through its role as a JAK phosphatase^31^. Structural variations in CD45 isoforms, including CD45RA, can alter its spatial organization and phosphatase activity, contributing to resistance mechanisms^32^. The expression and spatial behavior of surface proteins CD45 and CD82 in non-responders further support a disrupted membrane signaling architecture. Given the established role of CD82 in stem cell quiescence, chemotherapy resistance, and stabilization of CD45 isoforms, its polarization may play a critical role in the survival of leukemic cells both with and without FLT3 mutations^33–35^.

Our combinatorial drug screening revealed that SMAC mimetics are a promising class, showing robust synergy with midostaurin, particularly in CD38⁺CD45RA⁺ cells. In contrast, venetoclax, while clinically validated and effective in many AML contexts, did not show synergy with midostaurin in our assays and appeared to preferentially target CD34^hi^ cells. These findings suggest that the midostaurin/venetoclax combination may effectively reduce the bulk leukemic population but could leave behind resistant subsets that are less sensitive to Bcl-2 inhibition, such as CD38⁺CD45RA⁺ cells^9,10^. Rather than challenging current clinical strategies, our data provide complementary insights into the phenotypic diversity of AML, and the combination efficacy may depend on the cellular composition of the disease. Interestingly, it has been shown previously that the mechanism of SMAC-mimetics may be linked to TK receptor signaling, not limited to FLT3^36,37^. This could indicate a broader use for SMAC mimetics to overcome TKi resistance in other malignancies. Further studies are needed to validate the links between SMAC synergy and the non-responder cells, and to evaluate how phenotypic profiling can inform treatment selection and improve outcomes across heterogeneous patient populations.

While second-generation and more selective FLT3i are increasingly evaluated in clinical trials, our ex vivo analyses indicate that midostaurin and other earlier-generation FLT3i exhibit activity across a broader subset of patient samples compared to more selective FLT3i. Our concordance analysis demonstrates that midostaurin sensitivity is only weakly aligned with sensitivity to selective FLT3 inhibition, indicating that its ex vivo activity cannot be attributed solely to on-target FLT3 pathway dependence. In this study, we did not directly assess target engagement or kinase selectivity but instead focused on functional drug-response patterns within a precision medicine framework. From this perspective, compound-specific polypharmacology may contribute to broader ex vivo activity and could potentially be leveraged therapeutically, even when drugs are not designed to act exclusively on a single target^38^. This framework may also help explain why FLT3i, including those optimized for FLT3-ITD, can exhibit activity in FLT3-TKD or FLT3 wild-type samples. Accordingly, while midostaurin shows broader functional activity in this dataset, its mechanism of action appears context-dependent and influenced by both cellular phenotype and compound-specific properties. Further studies integrating functional profiling with direct measures of target engagement will be required to determine the relative contributions of on- and off-target effects across different FLT3i.

Finally, midostaurin demonstrated functional activity across both FLT3^mut^ and FLT3^wt^ samples in our ex vivo setting. These observations are consistent with the rationale underlying trials such as QuANTUM-WILD^39^ and suggest that phenotypic context may influence FLT3i response independently of direct FLT3 pathway activation. Together, these findings support a model in which functional drug sensitivity reflects an interplay between genetic background, cellular state, and compound-specific properties, rather than a singular dependence on FLT3 mutation status. Larger and clinically annotated cohorts will be required to determine whether cell state-based biomarkers can complement genetic profiling in guiding FLT3i use.

Limitations of the present study include the modest sample size of patients clinically treated with midostaurin, and validation of the predictability of functional testing in this context needs to be conducted in larger cohorts of patients. Further studies should determine the importance of membrane polarization and signaling shifts in STAT5 and AKT for inherent FLT3i resistance in CD38⁺CD45RA⁺ cells using purified cell populations and knock-down models. Additionally, the IAP dependency and proposed synergy mechanisms in resistant cells could be verified using methods such as BH3-profiling^40^. Due to the increase in clinical trials on other FLT3i, it would be essential to compare resistance mechanisms across different FLT3i and study the differences between FLT3i off-target effects more closely in both FLT3^mut^ and FLT3^wt^ cohorts.

In conclusion, our findings indicate that functional testing may contribute to understanding and potentially predicting clinical treatment response, consistent with trends observed in previous functional precision medicine studies. Furthermore, the results highlight the possible role of phenotypic and signaling context, alongside genetic factors, in influencing FLT3 inhibitor sensitivity, warranting further investigation in larger and clinically diverse cohorts. The identification of a cell state-linked resistant subset, together with the observed efficacy of SMAC mimetic combinations, may represent a potential strategy to overcome midostaurin resistance. These findings support further investigation into cell-state-informed therapeutic strategies and highlight the value of integrating molecular data with functional profiling to refine treatment selection in AML.

## Methods

### Leukemic cell isolation and thawing

PB or BM was collected from AML patients after obtaining written informed consent at the Karolinska University Hospital, Uppsala University Hospital, and through the acute leukemia biobank. The study adhered to the Declaration of Helsinki and received approval from the regional Ethical Review Authority in Stockholm (DNR: 2017/2085-31/2). BM-MNCs were isolated for AML samples at diagnosis via density centrifugation as described previously^41^. Biobanked cells were thawed according to the previously described protocol^41^.

### Cell lines

The AML cell lines PL-21 (ACC 536, DSMZ, Braunschweig, Germany), MOLM-13 (ACC-554), and MV4-11 (ACC-102) were cultured in RPMI 1640 with 2 mM L-glutamine, 100 IU/mL penicillin, 0.1 mg/mL streptomycin, and 10–20% FBS. The cell lines were mycoplasma tested and STR profiled using the Eurofins Human Cell Line Authentication Service.

### MS-proteomics

MS-proteomics was performed as described previously^41^. Briefly, cell pellets were lysed with 4% SDS lysis buffer and prepared with a modified SP3 protein cleanup and digestion protocol^42^. Peptides were labeled with TMT10-plex and separated by immobilized pH gradient-isoelectric focusing (IPG-IEF) on 3–10 strips as described previously^43^. Extracted IPG-IEF peptide fractions were separated using an online 3000 RSLCnano system coupled to a Thermo Scientific Q Exactive-HF. MSGF+ and Percolator in the Nextflow platform were used to match MS spectra to the Ensembl 92 human protein database^44^.

### Soluble proteomics

Supernatants were acquired from bone marrow samples diluted in RPMI medium after centrifugation at 300×*g* for 10 min and stored at −80 °C. Proximity Extension Assay (PEA) was performed on 1 μL of supernatant was diluted 1:1 according to manufacturer’s instructions using the Olink 96 Immuno-Oncology panel (Olink Proteomics, Uppsala, Sweden). Sample detection and analysis were done by real-time PCR analysis on the BioMarkTM HD System (Fluidigm, San Francisco, CA).

### RNA and panel DNA sequencing

RNA and DNA sequencing data from a previously published cohort were used^45^. Clinical parameters and treatment outcomes were acquired through patient records and the Swedish Adult Acute Leukemia Registry.

### Drug sensitivity and resistance testing

DSRT was performed as described previously^41^. In short, BM-MNCs were resuspended in complete RPMI (RPMI 1640, 10% FBS, 2 mM L-glutamine, 100 IU/mL penicillin, 0.1 mg/mL streptomycin, and 12.5% HS-5-conditioned media) and dispensed in pre-made drug plates (FIMM HTB)^13^. Incubation was done at 37 °C and 5% CO_2_. Cell viability was evaluated after 72 h by the addition of CellTiterGlo (CTG, Promega, Madison, WI, USA) and quantifying luminescence on an EnSight plate reader (PerkinElmer). Drug sensitivity scores (DSS) were then calculated with Breeze^46^, and healthy bone marrow control DSS were used to calculate the selective DSS (sDSS). For combination screening, midostaurin was added to the culture medium at the previously determined IC_50_ for the tested cell line or patient cells. For synergy screening, custom drug plates were made with an Echo 550 (Beckman Coulter, Brea, CA) with each drug combination in an 8 × 8 concentration matrix to test drug synergy. To ensure robust classification, each DSRT experiment underwent extensive assay-level quality control using established performance metrics, including *Z*′-factor benchmarking. Samples failing to meet quality criteria were excluded from analysis. The DSS and sDSS metrics integrate both potency and efficacy across the full dose–response curve and are robust to local variability and technical noise, supporting reliable identification of sensitive versus less-sensitive samples.

### Single-cell spatial proteomics

Six vitally frozen BM-MCNs, two of which were run in duplicate, were thawed and processed according to the manufacturer’s instructions of the Single Cell Spatial Proteomics Kit, Immunology Panel I (Pixelgen Technologies, Stockholm, Sweden)^18^. Afterwards, all samples were prepared and sequenced on a NovaSeq X Plus system on a 10B flow cell and XLEAP-SBS sequencing chemistry, with 15% PhiX spike-in, at the SciLifeLab National Genomics Infrastructure (Uppsala, Sweden) according to routine protocols.

### Flow cytometry

BM-MNCs were washed in FACS buffer (PBS, 0.5% BSA, and 2 mM EDTA) followed by FcX blocking as described previously^41^. Afterwards, antibodies were incubated for 30 min, 4 °C in FACS buffer, followed by several washing steps (Supplementary Table 4). Data was acquired on an Attune (Thermo Fisher).

### Phosphoflow

Following midostaurin stimulation, BM-MNCs and cell lines were incubated with Zombie Aqua live/dead dye for 10 min at 4 °C in FACS buffer, followed by fixation with 4% PFA for 10 min at 37 °C. Next, ice-cold BD Phosflow Perm Buffer III (BD Biosciences) was added for 30 min while cells were kept on ice. Afterwards, cells were washed twice, and blocking/staining was performed as described in the previous section.

### Statistics, data analysis, and visualization

Plots were generated with R (v.4.4.0) using RStudio (v.2023.12.1 + 402) with ggplot2 (v.3.5.1), ggrepel (v.0.9.5), cowplot (1.1.3), patchwork (v.1.2.0), and RColorBrewer (v.1.1.3) unless stated otherwise.

DSRT and clinical data heatmaps were generated with ComplexHeatmaps (v.2.20.0), circlize (v.0.4.16), and colorspace (v.2.1.0). Uni- and multivariate analyses were done using compareGroups (v.4.8.0) and stats (v.4.4.0) packages. Univariate *p* values were obtained with Fisher’s exact test and Mann–Whitney *U* test for categorical and continuous variables, respectively. Multivariate *p*-values were obtained using multivariate logistic regression analysis. For differential expression, a two-sample *t*-test with multiple testing correction was done using Benjamini–Hochberg (BH) false discovery rate (FDR) of 5%. PCA was done using FactoMineR (v.2.11). Drug screening curves were fitted with minipack.lm (v.1.2.4). Kaplan–Meier curves and log-rank tests were generated with survival (v.3.8-3) and survminer (v.0.5.0).

Molecular data gene set enrichment analysis (GSEA) was done with clusterProfiler (v.4.12.0) and msigdbr (v.7.5.1), using pre-ranked Log2FC values and an FDR of 5%. The hallmark gene sets from MSigDB were used, as well as sets generated by van Galen et al. ^15^ and Zeng et al. ^16^. Ridgeplots were generated with ggridges (v.0.5.6). Linear regression and Spearman’s rank correlation were done with protein/gene expression as the independent variable, and midostaurin sDSS as the dependent variable.

For the Soluble proteomic data, ct-data from the Fluidigm Real-Time PCR Analysis software (Fluidigm) was analyzed and normalized with the NPX Manager analysis software (Olink) based on internal and external assay controls, resulting in Normalized Protein eXpression (NPX) values, a log2-scale relative quantification.

Single-cell spatial proteomic (MPX) raw fastq data files were processed with the nf-core/pixelator pipeline (v.0.15.2), where low-quality reads were removed and matched against the common pixel binding motifs^18^. The generated pixel files contain counts and spatial information of all proteins in each cell. Downstream analysis was done in R using pixelatorR (v.0.4.0). Data were first filtered for low-quality cells, centered log-ratio transformed, and antibody markers with a low median expression and isotype controls were removed. Graph-based clustering was done using the Louvain algorithm for community detection, applied to a shared nearest neighbor (SNN) graph based on the first 10 principal components. Clusters were annotated by their surface expression profiles, and B and T cell clusters were removed before re-clustering with only myeloid cells. UMAP was used to visualize the data. A two-sided Wilcoxon rank-sum test was done to determine the most differentially expressed surface markers, with an FDR of 5%. Differential polarity and co-localization scores were then calculated. Co-localization was visualized with the Fruchterman–Reingold (FR) force-directed layout algorithm for two representative cells based on the UPIA and UPIB pixel identifiers.

Flow cytometric data were gated in Flowjo (BD Biosciences) for live cells and exported to R. Arcinsh transformation was done with cofactor 5 using flowCore (v.2.16.0), and an expression matrix was extracted. Data was scaled with matrixStats (v.1.3.0) from 0 to 1 for visualization purposes in the histograms, which were generated with reshape2 (v.1.4.4). The expression matrix was then clustered using FlowSom (v.2.12.0) and ConsensusClusterPlus (v.1.68.0) and visualized with pheatmap (v.1.0.12). Dimensionality reduction was performed with Rtsne (v.0.17). Umaps were generated with umap (v.0.2.10.0).

Drug synergy was calculated with synergyfinder (v.3.12.0). Sequential drug screening curves were fitted with minipack.lm (v.1.2.4).

## Supplementary information

Supplementary Figures

Supplementary data

## Data Availability

Normalized transcriptomic data have been published previously as part of the Clinseq-AML cohort and are publicly available (10.5281/zenodo.292986). Drug testing, soluble proteomics, and spatial proteomics data sets are not publicly available due to privacy reasons and are stored at the Swedish National Data Service (SND-ID: 2025-057, Version 1, 10.48723/n3h9-wr71 and SND-ID: 2025-041, Version 1, 10.48723/fjzh-zd37). MS-proteomics data will be publicly available upon publication of the original study, which generated the data (PRIDE identifier: PXD061758). Contact Professor Janne Lehtiö (janne.lehtio@ki.se) for more details.

## Code availability

The full R analysis code is available on GitHub (https://github.com/nstruyf/FLT3_SMAC).
