# Supplementary material for: Midostaurin response in AML is shaped by a progenitor-like cell state selectively targeted by SMAC mimetics
Source: NPJ Precis Oncol. 2026 Mar 11;10:117. doi: 10.1038/s41698-026-01363-8 (PMC12996285; doi:10.1038/s41698-026-01363-8)
Supplement: Supplementary file 1 — Supplementary Figures [file 41698_2026_1363_MOESM1_ESM.pdf]

Midostaurin response in AML is shaped by a progenitor-like cell state selectively targeted by SMAC mimetics

Supplementary Figures

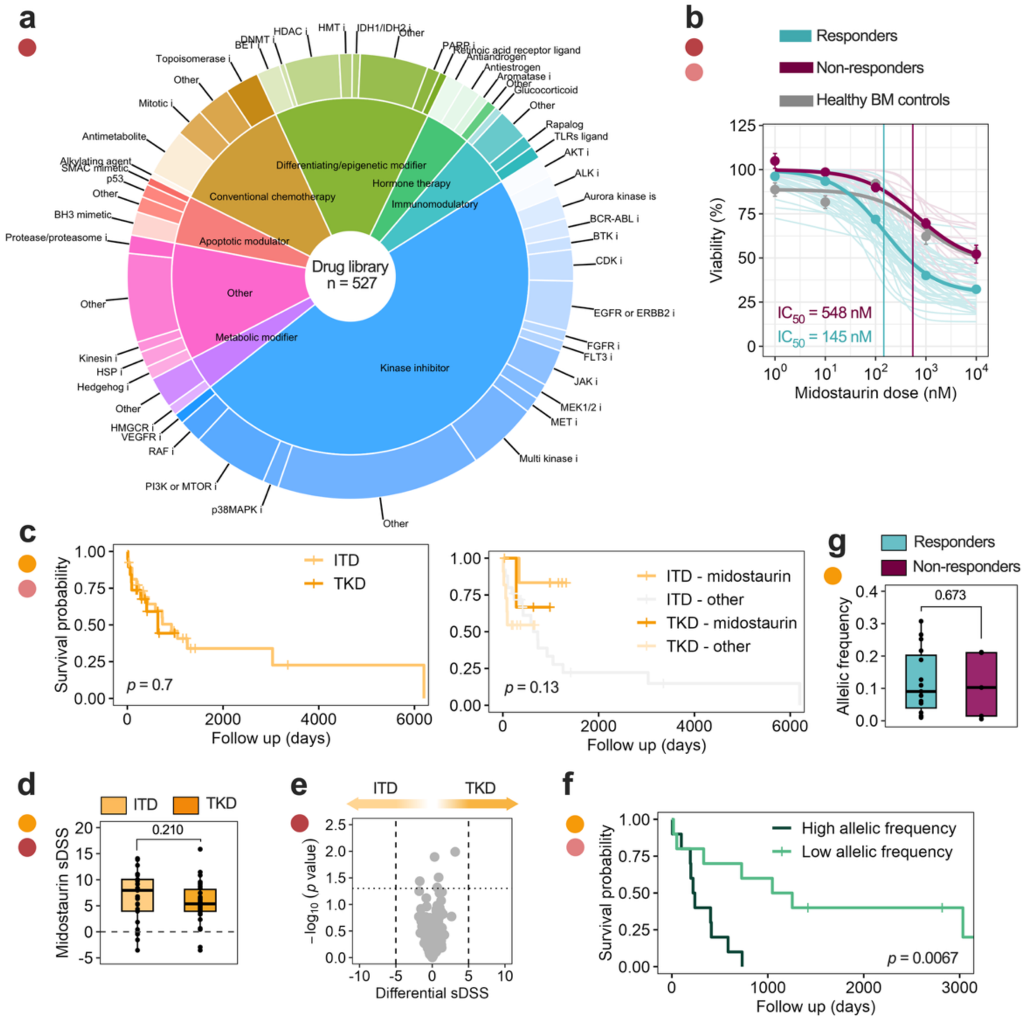

Supplementary figure 1: (a) Overview of drug class and subclass distribution in the tested drug library. (b) Individual midostaurin drug response curves for all patients (n = 63) with mean IC_{50} for each group, and healthy BM controls (n = 3), shown with mean and SEM. (c) Kaplan-Meier curves for all FLT3-TKD- (n = 19) and -ITD (n = 27) patients (left), and for those treated with midostaurin (TKD: n = 3, ITD: n = 7) or other (TKD: n = 11, ITD: n = 25) therapies (right). (d) Midostaurin sDSS comparison between TKD- (n = 30) and ITD patients (n = 33). (e) Differential sDSS of all tested drugs between TKD- and ITD patients, done with multiple t-tests using an FDR of 5% and showing drugs with p < 0.05 and a differential sDSS > 5. (f) Five-year survival curve for all $FLT3^{mut}$ patients with high (n = 10) or low allelic frequency (n = 10). (g) Comparison of allelic ratio of FLT3-ITD between responders (n = 14) and non-responders (n = 6). Statistical significance was assessed with the Mann-Whitney U test for paired comparisons and Mantel-Cox test for Kaplan-Meier curves.

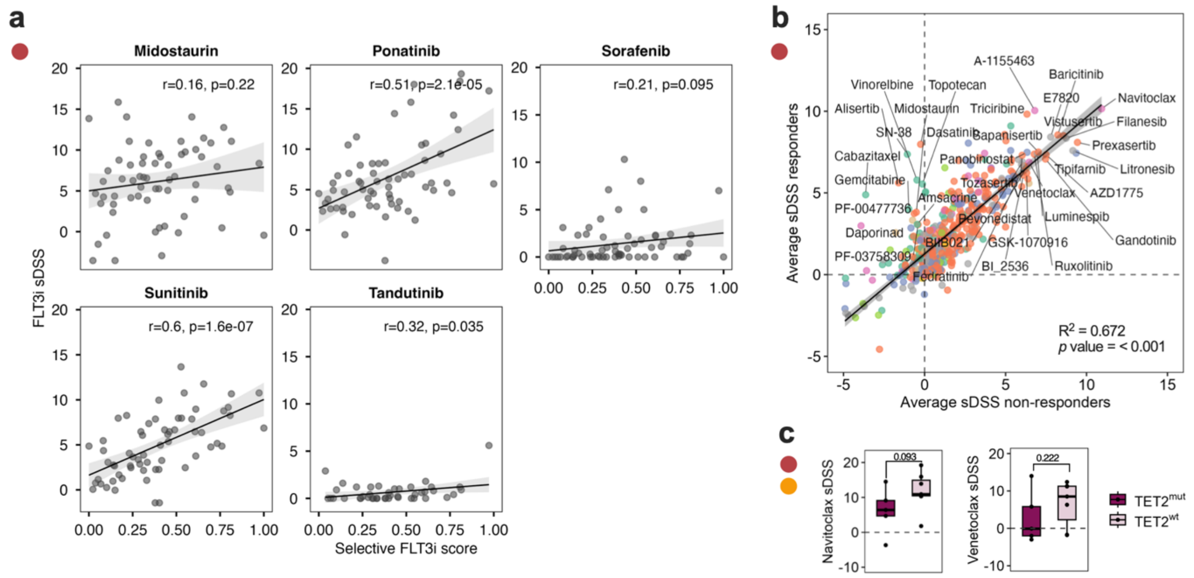

Supplementary Figure 2: (a) Scatter plots showing the relationship between the selective FLT3i sensitivity score (calculated as the mean sDSS across selective FLT3i gilteritinib, quizartinib, and crenolanib) and midostaurin or other broad-spectrum FLT3i sDSS for all tested patients (n = 63). (b) A comparison of average sDSS for all tested drugs (n =527) between responders (n = 49) and non-responders (n = 14), labels indicate drugs with an sDSS > 6 for both groups, shown with a linear regression model. (c) Boxplots showing difference in sDSS of selected drugs between TET2^{mut} (n = 5) and TET2^{wt} (n = 9) non-responders, statistical significance was assessed with the Mann-Whitney U test.

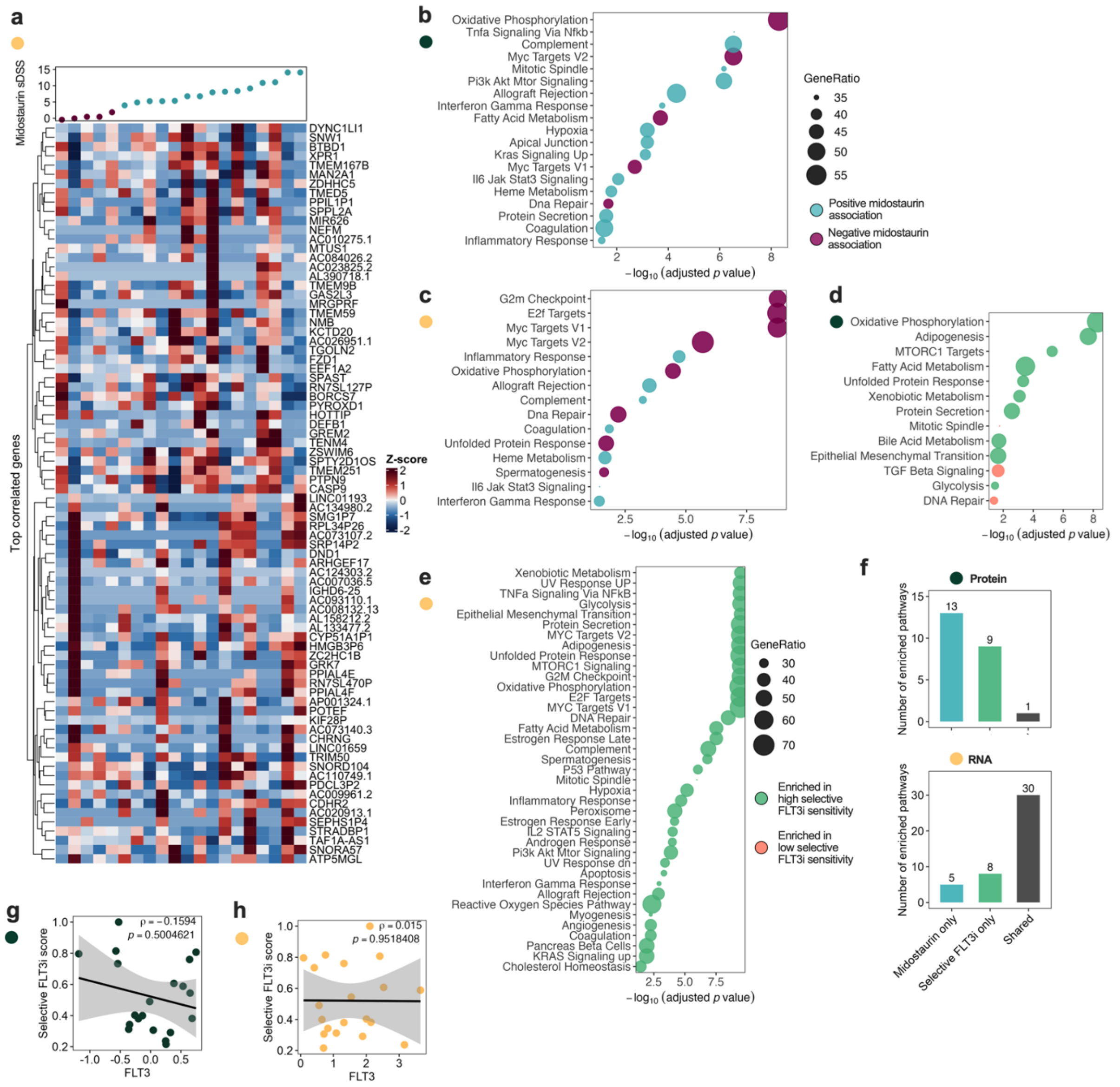

Supplementary Figure 3: (a) A heatmap of the top 40 most positively and negatively significantly ($p < 0.05$) correlated genes with midostaurin. (b-c) GSEA on Spearman's rank correlation with midostaurin response for protein (b) and RNA (c), showing enrichment distributions of each gene set and adjusted p values. (d-e) GSEA on Spearman's rank correlation with the selective FLT3i sensitivity score for protein (d) and RNA (e), showing enrichment distributions of each gene set and adjusted p values. (f) Comparison of proteomic and transcriptomic pathway overlap between the midostaurin and selective FLT3i pathway correlations. (g-h) Scatter plots showing the correlation of the selective FLT3i score with FLT3 protein (g) and RNA (h) levels, shown with a linear regression model.

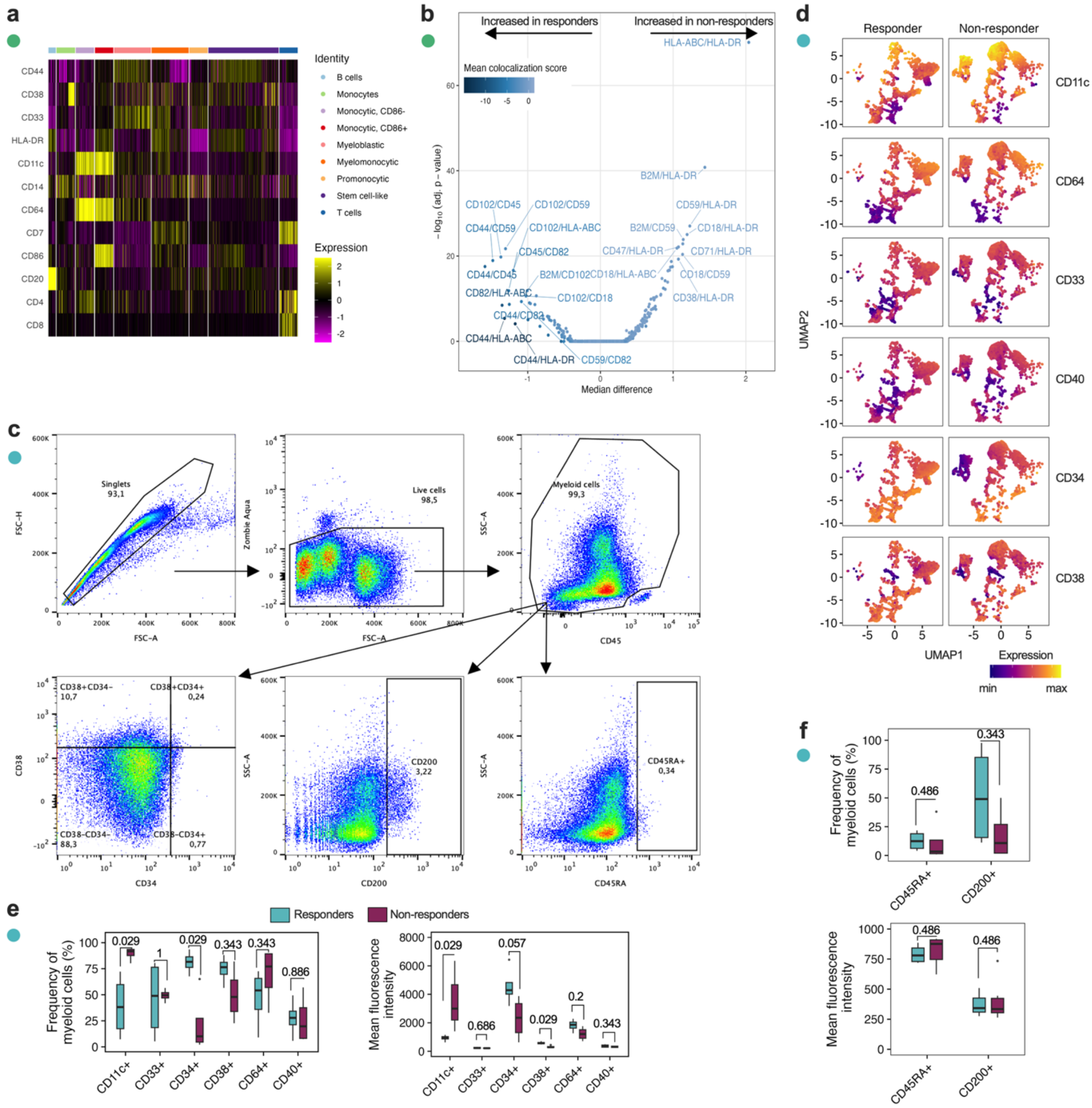

Supplementary Figure 4: (a) Heatmap of markers used for single-cell cluster annotation, shown as relative marker expression. (b) Differential co-localization of surface markers, showing markers colored by mean colocalization score with a significance cutoff (p < 0.05). (c) Gating strategy for flow cytometry assays. (d) UMAPs of expression for all markers measured in flow cytometry validation assay. (e) Frequency of myeloid cells and mean fluorescence intensity for all markers measured in flow cytometry validation assay. (f) Frequency of myeloid cells and mean fluorescence intensity for CD34/CD38 fractions measured in flow cytometry validation assay. Statistical significance was assessed with the Mann-Whitney U test.

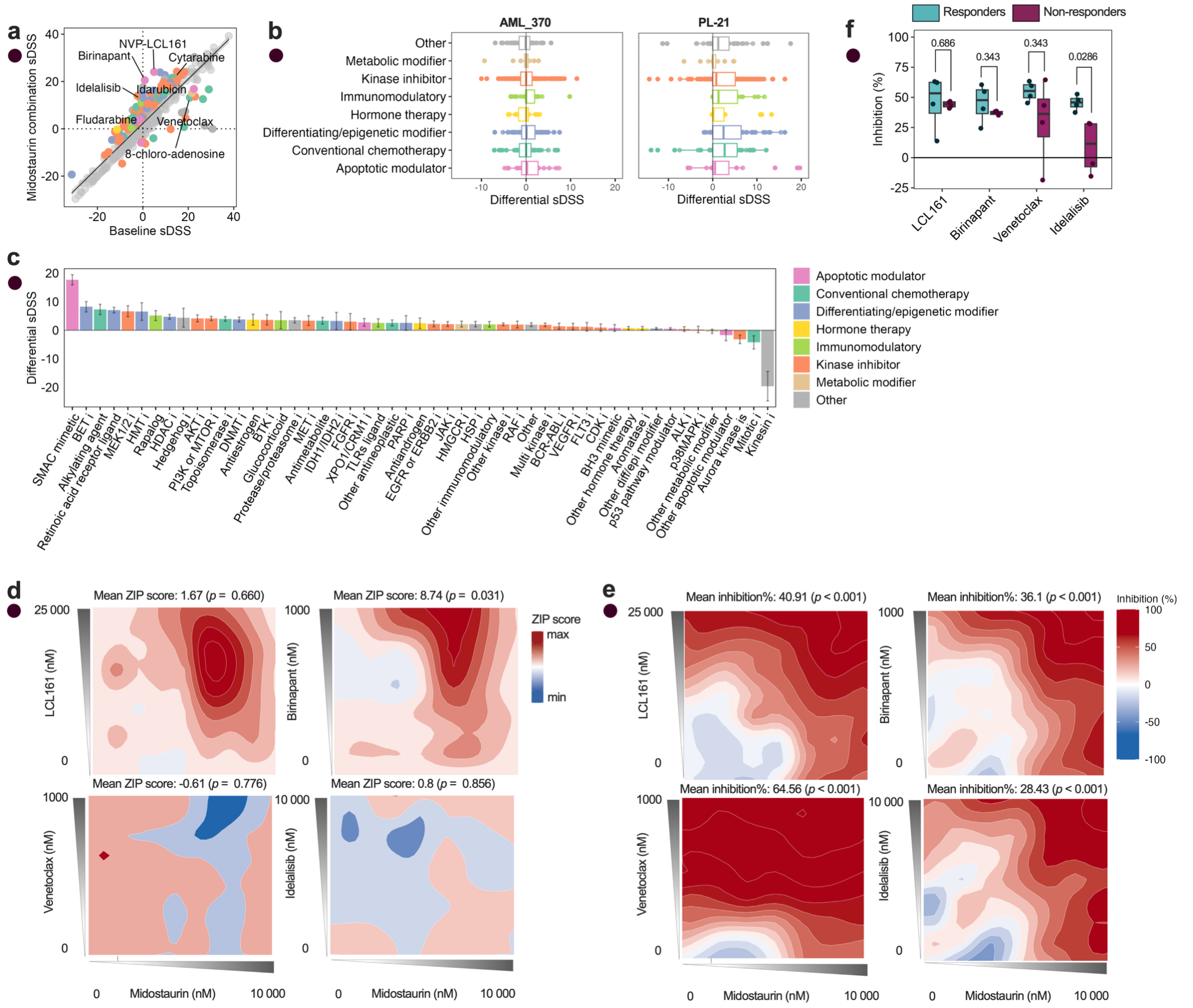

Supplementary Figure 5: (a) Combination screening on non-responder FLT3^{mut} cell line PL-21 displaying the selective drug sensitivity score (sDSS) for all tested drugs (n = 526) with the baseline sDSS on the x-axis and combination with 1200 nM midostaurin on the y-axis. (b) An overview of differential sDSS distribution based on drug class in the combination drug screening of a non-responder patient and PL-21. (c) A waterfall plot with the differential sDSS for all subclasses tested during combination screening of PL21, differential sDSS was calculated by retracting baseline sDSS from combination sDSS, shown as subclass mean with SD and ranked by mean. (d) Synergy screening ZIP scores of midostaurin with LCL161, birinapant, venetoclax and idelalisib in PL-21, shown with mean ZIP score for all. (e) Synergy screening % inhibition shown for one representative non-responder patient. (f) Synergy screening results for all responders (n = 4) and non-responders (n = 4) showing mean % inhibition for all tested combinations.

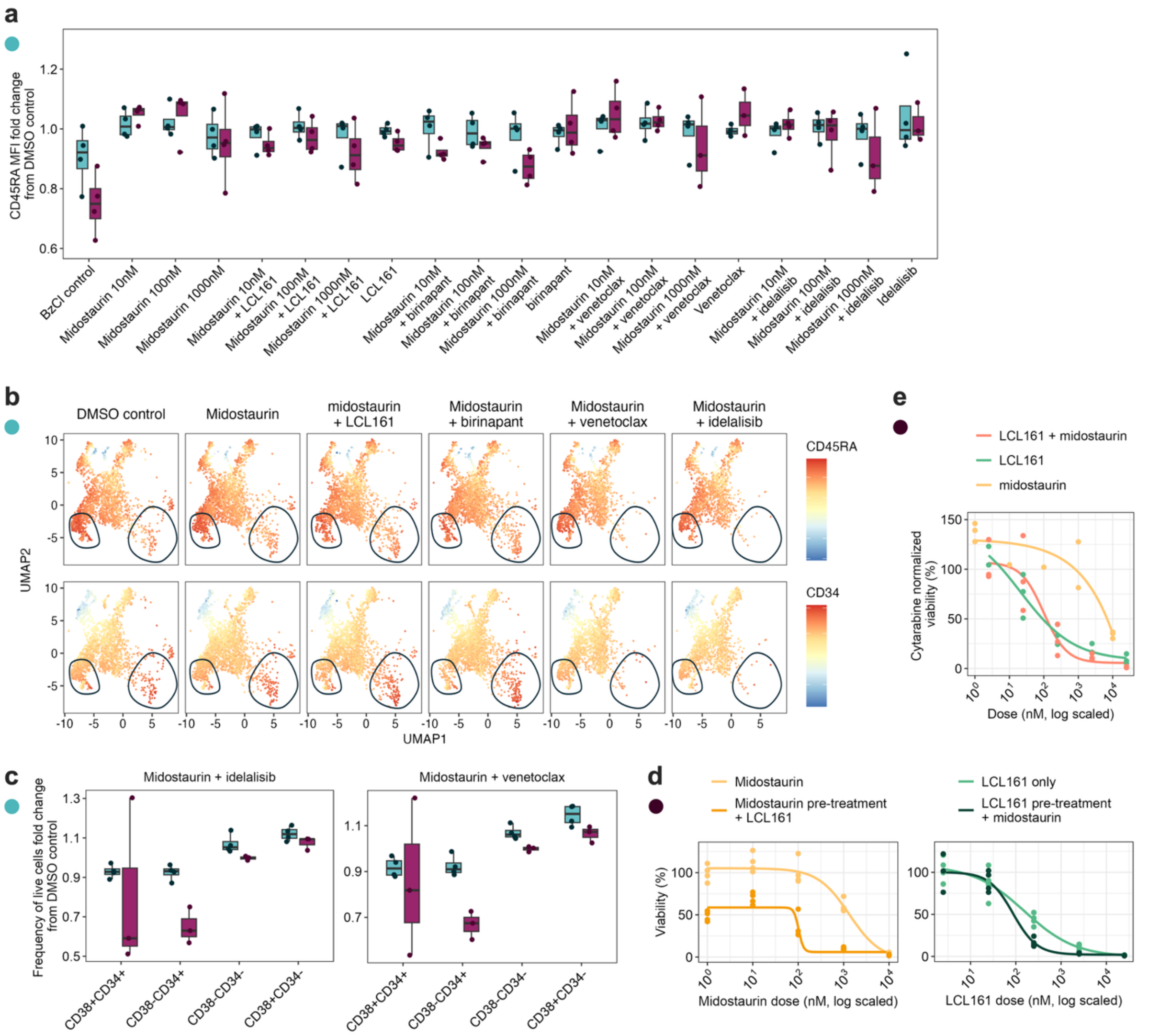

Supplementary Figure 6: (a) Boxplots showing CD45RA MFI fold change from DMSO control after 72h treatment with midostaurin alone or in combination with selected drugs, measured by flow cytometry in responders (n = 4) and non-responders (n = 4). (b) UMAPs showing CD45RA and CD38 expression in non-responder (n = 4) cells for midostaurin (1000 nM) alone or in combination with LCL161 (100 nM), birinapant (100 nM), Venetoclax (10 nM), and idelalisib (100 nM). (c) Boxplots showing frequency of live cell fold change from DMSO control for each CD34/CD38 fraction after 72h treatment with midostaurin un combination with idelalisib and venetoclax. (d) Sequential drug treatment of midostaurin (1200 nM) and LCL161 (480 nM) for 24h followed by 5-dose response of the other drug for 48h in PL21, performed in quadruplicate. (e) Sequential drug treatment of 48h cytarabine (100nM) treatment followed by a 5-dose treatment with LCL161, midostaurin or a combination of both (48h), normalized for cytarabine treatment viability and performed in quadruplicate.

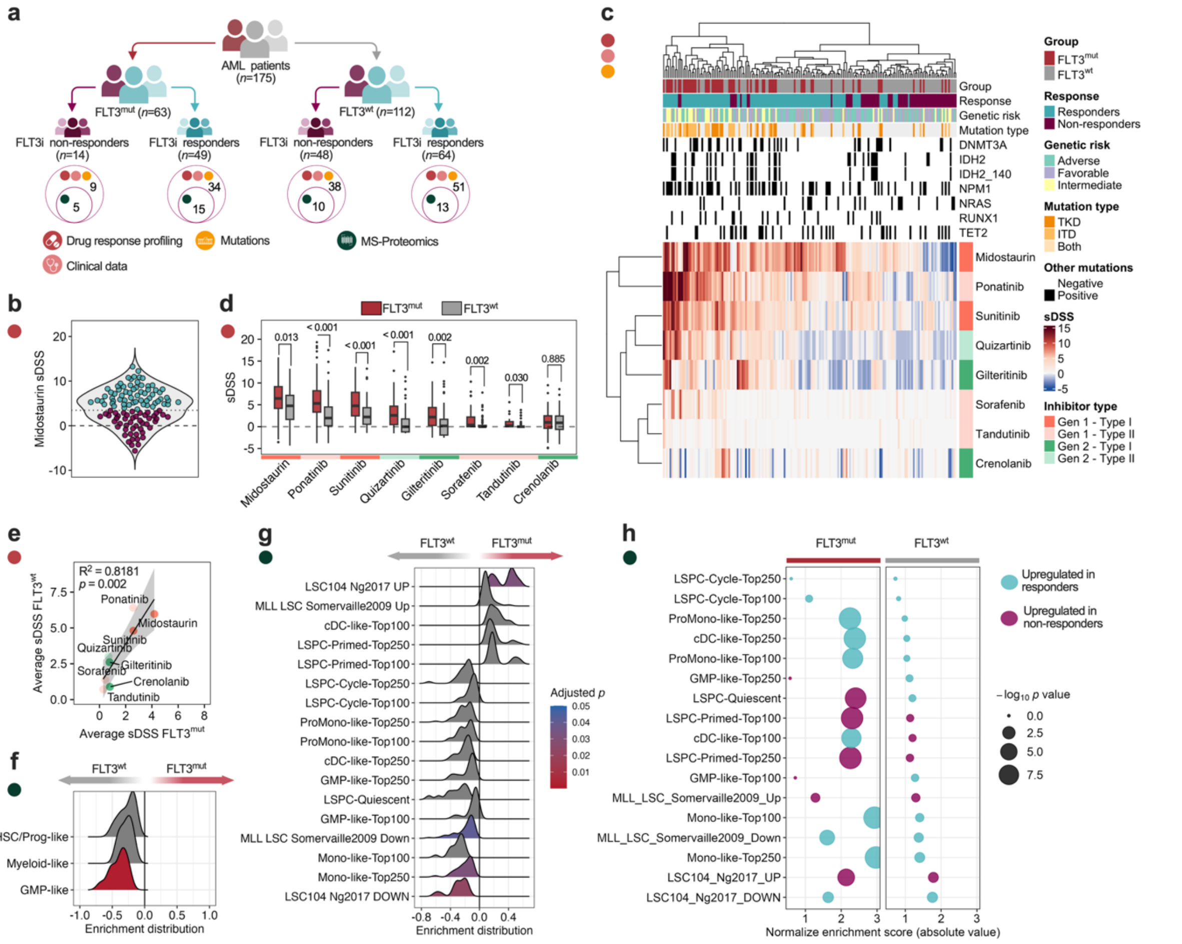

Supplementary Figure 7: Midostaurin sensitivity is higher in FLT3^{mut} AML and is linked to cellular differentiation independent of FLT3 mutation status. (a) Overview of the data overlap between patient groups. (b) *Ex vivo* midostaurin selective drug sensitivity score (sDSS) for all FLT3^{wt} patients (n = 112), showing the previously established threshold of 3.46 (dashed line) dividing patients into responders (n =64) and non-responders (n = 48). (c) Heatmap with FLT3i response in FLT3^{wt} (n = 112) FLT3^{mut} (n = 63) patients and the most common co-mutations, with hierarchical clustering performed on sDSS values. (d) Boxplots showing sDSS values for all FLT3i in FLT3^{mut} and FLT3^{wt} patients ordered by the heatmap clustering, statistical significance was assessed using the student's t-test. (e) XY plot showing average sDSS for all FLT3i in both groups colored by generation and inhibitor type, with a linear regression model. (f-g) GSEA of MS-proteomics data on FLT3^{wt} (n = 20) FLT3^{mut} (n = 20) patients using AML specific gene sets showing enrichment distributions of each gene set and adjusted p values. (h) GSEA of AML specific gene sets of FLT3^{wt} responders (n = 13) and non-responders (n = 10) compared to FLT3^{mut} responders (n = 11) and non-responders (n = 6), displaying the absolute normalized enrichment score (NES) on the x axis with size indicating -log10 p value.

Supplementary Tables:

Supplementary Table 1: Clinical parameters and cytogenetic characteristics of FLT3^{mut} midostaurin non-responders and responders.

	Non-responder (n=14)	Responder (n=49)	P-value (univariate)	P-value (multivariate)
**Sex (male)**	6 (50.0%)	24 (53.3%)	1	0.728
**Age (median, range)**	66.5 (39-84)	64 (23-80)	0.488	0.349
**AHD-AML***	0 (0.00%)	3 (6.52%)	1	0.997
**Bone marrow blasts (median, range)**	67.25 (35-98)	57 (16-100)	0.529	.
**WBC count (median, range)**	38.8 (14.2-374.3)	37.7 (0.58-245.9)	0.708	0.781
**ELN 2022:**			0.389	.
**Adverse**	2 (18.2%)	12 (29.3%)	.	.
**Favorable**	0 (0.00%)	5 (12.2%)	.	.
**Intermediate**	9 (81.8%)	24 (58.5%)	.	.
**Normal karyotype**	8 (57.1%)	37 (75.5%)	0.197	0.767
**Complex karyotype**	0 (0.00%)	0 (0.00%)	.	.
**t(15;17)**	0 (0.00%)	0 (0.00%)	.	.
**CBF AML****	0 (0.00%)	0 (0.00%)	.	.
**del(5q)/-5 or del(7q)/-7**	0 (0.00%)	1 (2.04%)	1	.
**Other*****	0.00 (0.00)	0.04 (0.20%)	0.159	.

Footnotes: *AHD-AML includes therapy related AML and secondary AML. **CBF-AML including t(8;21), inv(16) and t(16;16).*** Other cytogenetic aberrations include inv(3), t(3;3) and 11q23 abnormalities. Abbreviations: AHD-AML: AML with antecedent hematological disease; CBF-AML: Core binding factor AML; WBC: White blood cell.

Supplementary Table 2: Mutational characteristic of FLT3^{mut} midostaurin non-responders and responders.

	Non-responder (n=14)	Responder (n=49)	P-value (univariate)	P-value (multivariate)
**ASXL1:**	0 (0.00%)	2 (4.08%)	1	.
**CEBPA (single)**	0 (0.00%)	5 (10.2%)	0.578	0.994
**CEBPA (double**	0 (0.00%)	0 (0.00%)	.	.
**DNMT3A**	3 (21.4%)	18 (36.7%)	0.35	0.055
**EZH2**	0 (0.00%)	1 (2.04%)	1	.
**FLT3-TKD**	6 (42.9%)	24 (49.0%)	1	0.919
**FLT3-ITD**	9 (64.3%)	26 (53.1%)	1	0.660
**IDH1**	0 (0.00%)	1 (2.04%)	1	.
**IDH2**	2 (14.3%)	13 (26.5%)	0.486	.
**IDH2 140**	2 (14.3%)	11 (22.4%)	0.716	0.304
**IDH2 172**	0 (0.00%)	0 (0.00%)	.	.
**KIT**	0 (0.00%)	0 (0.00%)	.	.
**KRAS**	0 (0.00%)	0 (0.00%)	.	.
**NPM1**	10 (71.4%)	31 (63.3%)	0.753	0.309
**NRAS**	0 (0.00%)	5 (10.2%)	0.578	0.995
**PHF6**	0 (0.00%)	0 (0.00%)	.	.
**PTPN11**	0 (0.00%)	1 (2.04%)	1	.
**RAD21**	2 (14.3%)	1 (2.04%)	0.121	.
**RUNX1**	2 (14.3%)	7 (14.3%)	1	0.542
**SF3B1**	1 (7.14%)	1 (2.04%)	0.398	.
**SMC1A**	0 (0.00%)	2 (4.08%)	1	.
**SMC3**	0 (0.00%)	2 (4.08%)	1	.
**STAG2**	1 (7.14%)	2 (4.08%)	0.536	.
**TET2**	6 (42.9%)	8 (16.3%)	0.063	0.062
**TP53**	0 (0.00%)	0 (0.00%)	.	.
**U2AF1**	0 (0.00%)	1 (2.04%)	1	.
**WT1**	2 (14.3%)	7 (14.3%)	1	0.652

Supplementary Table 3: Multivariate logistic regression analyses of $FLT3^{mut}$ midostaurin non-responders and responders.

	Odds ratio	95% CI	P-value
**Sex (male vs female)**	1.35	0.23-8.08	0.728
**Age (continuous)**	0.96	0.88-1.03	0.349
**De novo* (yes vs no)**	0.00	.	0.997
**WBC count (continuous)**	1	0.99-1.01	0.781
**Normal karyotype (yes vs no)**	0.73	0.07-5.5	0.767

Footnotes: *AHD-AML includes therapy related AML and secondary AML. **CBF-AML including t(8;21), inv(16) and t(16;16).*** Other cytogenetic aberrations include inv(3), t(3;3) and 11q23 abnormalities. Abbreviations: AHD-AML: AML with antecedent hematological disease; CBF-AML: Core binding factor AML; WBC: White blood cell

Supplementary Table 4: Flow cytometry antibody list

Fluorophore	Marker	Clone	Manufacturer	Dilution	Catalog number
PE	CD11c	B-ly6	BD biosciences	1:40	555392
FITC	CD33	HIM3-4	BD biosciences	1:40	555626
APC	CD34	8G12	BD biosciences	1:40	345804
BV421	CD38	HIT2	BD biosciences	1:40	562444
PE-Cy7	CD40		BD biosciences	1:40	561215
BV786	CD45	HI30	BD biosciences	1:40	563716
RB780	CD45RA	HI100	BD biosciences	1:20	569082
APC-H7	CD64	10.1	BD biosciences	1:40	561190
PE	CD200	MRC OX-104	BD biosciences	1:5	552475
RY586	STAT5 (pY694)	47/Stat5(pY694)	BD biosciences	1:20	568145
BV421	ERK1/2 (pT202/pY204)	20A	BD biosciences	1:20	562981
Alexa Fluor 647	Akt (pS473)	M89-61	BD biosciences	1:5	560343
Zombie Aqua	Live cells	-	Biolegend	1:200	423102

Supplementary Table 5: Clinical parameters and cytogenetic characteristics of FLT3^{mut} vs FLT3^{wt} patients.

	**FLT3^{mut} (n=63)**	**FLT3^{wt} (n=112)**	**P-value (univariate)**	**P-value (multivariate)**
**Sex (male)**	30 (52.6%)	66 (60.0%)	0.454	0.728
**Age (median, range)**	66 (23-84)	64 (26-89)	0.701	0.349
**AHD-AML***	3 (5.08%)	19 (17.1%)	0.009	0.997
**Bone marrow blasts (median, range)**	60 (16-100)	46 (0-96.6)	0.013	.
**WBC count (median, range)**	37.7 (0.58-374.3)	24.25 (0.2-473.6)	0.31	0.781
**ELN 2022:**			<0.001	.
**Adverse**	14 (26.9%)	47 (50.5%)		
**Favorable**	5 (9.62%)	41 (44.1%)		
**Intermediate**	33 (63.5%)	5 (5.38%)		
**Normal karyotype**	45 (71.4%)	35 (31.5%)	<0.001	0.767
**Complex karyotype**	0 (0.00%)	23 (20.7%)	<0.001	.
**t(15;17)**	0 (0.00%)	1 (0.90%)	1	.
**CBF AML****	1 (1.59%)	13 (11.7%)	0.038	.
**del(5q)/-5 or del(7q)/-7**	1 (1.59%)	23 (20.7%)	0.001	.
**Other*****	0.03 (0.18)	0.22 (0.42)	<0.001	.

Footnotes: *AHD-AML includes therapy related AML and secondary AML. **CBF-AML including t(8;21), inv(16) and t(16;16).*** Other cytogenetic aberrations include inv(3), t(3;3) and 11q23 abnormalities. Abbreviations: AHD-AML: AML with antecedent hematological disease; CBF-AML: Core binding factor AML; WBC: White blood cell.

Supplementary Table 6: Mutational characteristic of $FLT3^{mut}$ vs $FLT3^{wt}$ patients.

	$FLT3^{mut}$ (n=63)	$FLT3^{wt}$ (n=112)	P-value (univariate)	P-value (multivariate)
**ASXL1:**	2 (3.17%)	15 (13.4%)	0.054	.
**CEBPA (single)**	5 (7.94%)	7 (6.25%)	0.758	0.107
**CEBPA (double**	0 (0.00%)	3 (2.68%)	0.554	.
**DNMT3A**	21 (33.3%)	17 (15.2%)	0.009	0.315
**EZH2**	1 (1.59%)	5 (4.46%)	0.421	.
**FLT3-TKD**	30 (47.6%)	0 (0.00%)	<0.001	0.104
**FLT3-ITD**	35 (55.6%)	0 (0.00%)	<0.001	0.450
**IDH1**	1 (1.59%)	7 (6.25%)	0.261	.
**IDH2**	15 (23.8%)	17 (15.2%)	0.225	.
**IDH2 140**	13 (20.6%)	12 (10.7%)	0.115	0.281
**IDH2 172**	0 (0.00%)	4 (3.57%)	0.298	.
**KIT**	0 (0.00%)	7 (6.25%)	0.05	.
**KRAS**	0 (0.00%)	6 (5.36%)	0.089	.
**NPM1**	41 (65.1%)	19 (17.0%)	<0.001	0.213
**NRAS**	5 (7.94%)	17 (15.2%)	0.25	0.434
**PHF6**	0 (0.00%)	2 (1.79%)	0.537	.
**PTPN11**	1 (1.59%)	5 (4.46%)	0.421	.
**RAD21**	3 (4.76%)	3 (2.68%)	0.668	.
**RUNX1**	9 (14.3%)	10 (8.93%)	0.401	0.592
**SF3B1**	2 (3.17%)	1 (0.89%)	0.294	.
**SMC1A**	2 (3.17%)	3 (2.68%)	1	.
**SMC3**	2 (3.17%)	0 (0.00%)	0.128	.
**STAG2**	3 (4.76%)	8 (7.14%)	0.748	.
**TET2**	14 (22.2%)	24 (21.4%)	1	0.388
**TP53**	0 (0.00%)	12 (10.7%)	0.005	.
**U2AF1**	1 (1.59%)	1 (0.89%)	1	.
**WT1**	9 (14.3%)	4 (3.57%)	0.015	0.969

Supplementary Table 7: Multivariate logistic regression analyses of $FLT3^{mut}$ vs $FLT3^{wt}$ patients.

	Odds ratio	95% CI	P-value
**Sex (male vs female)**	1.08	0.46-2.52	0.858
**Age (continuous)**	0.98	0.95-1.01	0.218
**De novo* (yes vs no)**	0.46	0.11-1.61	0.252
**WBC count (continuous)**	1.00	0.99-1.00	0.230
**Normal karyotype (yes vs no)**	2.36	0.92-6.40	0.079

Footnotes: *AHD-AML includes therapy related AML and secondary AML. **CBF-AML including t(8;21), inv(16) and t(16;16).*** Other cytogenetic aberrations include inv(3), t(3;3) and 11q23 abnormalities. Abbreviations: AHD-AML: AML with antecedent hematological disease; CBF-AML: Core binding factor AML; WBC: White blood cell

Supplementary Data:

Supplementary Data 1: Drug library overview.

Supplementary Data 2: Dose response data for all 63 patients including response status and sDSS.

Supplementary Data 3: LSPC gene set created by Zeng et al.

Supplementary Data 4: List of proteins significantly correlated to *ex vivo* midostaurin response.

Supplementary Data 5: List of transcriptomic genes significantly correlated to *ex vivo* midostaurin response.

Supplementary Data 6: List of 76 surface markers used for spatial single-cell proteomics.
